# Evolutionary Game Theory and Social Learning Can Determine How Vaccine Scares Unfold

**DOI:** 10.1371/journal.pcbi.1002452

**Published:** 2012-04-05

**Authors:** Chris T. Bauch, Samit Bhattacharyya

**Affiliations:** 1 Department of Mathematics and Statistics, University of Guelph, Guelph, Canada; 2 Department of Ecology and Evolutionary Biology, Princeton University, Princeton, New Jersey, United States of America; 3 Department of Mathematics, University of Utah, Salt Lake City, Utah, United States of America; Pennsylvania State University, United States of America

## Abstract

Immunization programs have often been impeded by vaccine scares, as evidenced by the measles-mumps-rubella (MMR) autism vaccine scare in Britain. A “free rider” effect may be partly responsible: vaccine-generated herd immunity can reduce disease incidence to such low levels that real or imagined vaccine risks appear large in comparison, causing individuals to cease vaccinating. This implies a feedback loop between disease prevalence and strategic individual vaccinating behavior. Here, we analyze a model based on evolutionary game theory that captures this feedback in the context of vaccine scares, and that also includes social learning. Vaccine risk perception evolves over time according to an exogenously imposed curve. We test the model against vaccine coverage data and disease incidence data from two vaccine scares in England & Wales: the whole cell pertussis vaccine scare and the MMR vaccine scare. The model fits vaccine coverage data from both vaccine scares relatively well. Moreover, the model can explain the vaccine coverage data more parsimoniously than most competing models without social learning and/or feedback (hence, adding social learning and feedback to a vaccine scare model improves model fit with little or no parsimony penalty). Under some circumstances, the model can predict future vaccine coverage and disease incidence—up to 10 years in advance in the case of pertussis—including specific qualitative features of the dynamics, such as future incidence peaks and undulations in vaccine coverage due to the population's response to changing disease incidence. Vaccine scares could become more common as eradication goals are approached for more vaccine-preventable diseases. Such models could help us predict how vaccine scares might unfold and assist mitigation efforts.

## Author Summary

“Herd immunity” is a phenomenon whereby an entire population—including unvaccinated individuals—can be protected from infection by vaccinating only a certain percentage of the population. This suggests that immunization programs can be victims of their own success: past vaccinations can drive disease incidence to such low levels that as-yet unvaccinated individuals feel no incentive to get vaccinated, which creates conditions for future outbreaks. “Behavior-incidence” models capture this interplay between disease dynamics and vaccinating behavior. However, the predictive and explanatory value of these models is rarely tested against empirical data, and it is not clear whether the implied strategic interaction between individuals drives vaccinating behavior in real populations. Here we develop a behavior-incidence model based on evolutionary game theory and social learning. We show it often explains vaccine coverage data during a vaccine scare better than most competing models without strategic interactions and/or social learning. It can also predict future vaccine coverage and disease incidence peaks to a significant extent. Thus, strategic interactions between individuals via herd immunity appear to be a significant driver of behavior during a vaccine scare. It may be possible to harness behavior-incidence models to predict how future vaccine scares might unfold and possibly also to mitigate them.

## Introduction

**Figure 1:**
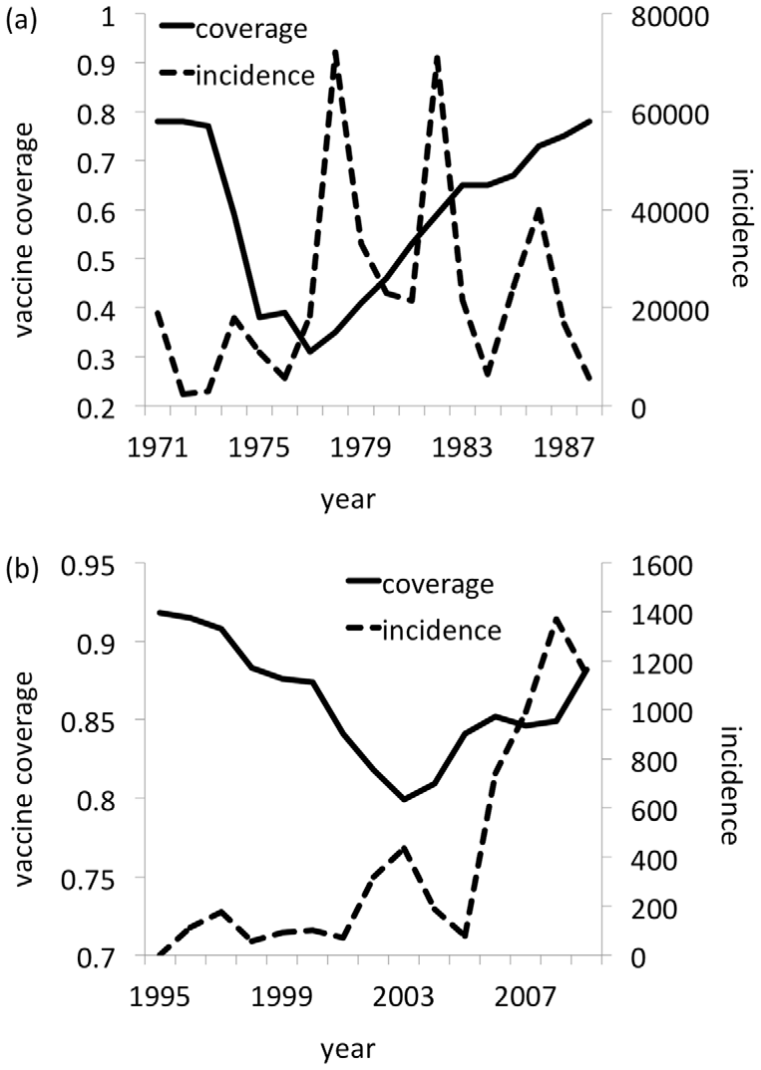
Whole cell pertussis vaccine coverage (solid line) and pertussis case notifications (dashed line), England & Wales 1971–1988 [**27**] (a); measles-mumps-rubella vaccine coverage (solid line) and lab-confirmed measles case notifications (dashed line), England & Wales 1995–2009 [**27**] (b). Media reports of alleged vaccine risks began in 1974 for pertussis and 1998 for MMR [15], [16]. Vaccine coverage is defined as percentage completing their primary courses by their second birthday. Hence, to correct for the ambiguity in precise age of vaccination in the model and allow comparison with case notification data, the vaccine coverage data in this figure are shifted by one year.

Vaccine coverage in England & Wales during the whole cell pertussis vaccine scare in the 1970s and the measles-mumps-rubella (MMR) vaccine scare in the 1990s share a common pattern of decline and recovery over many years (Figure 1). For pertussis, the decline resulted in large-scale outbreaks. MMR coverage declined much less and the resulting outbreaks were smaller, although measles was declared endemic again by 2008 [1].

Theory suggests that vaccine scares exemplify a “free-rider problem”: vaccine-generated herd immunity can reduce disease incidence to such low levels that vaccine risks appear large in comparison, causing some individuals to cease vaccinating. Hence, these non-vaccinators effectively “free ride” on the herd immunity generated by vaccinators. Game theory analyzes situations where the outcome of an individual's choice depends on the choices made by other individuals. Thus, game theory can be used to analyze free-rider problems such as vaccine scares. A growing literature combines mathematical models of disease transmission with game theory or other behavioral models to explore the feedback loop that connects disease incidence and vaccinating behavior among individuals: disease incidence influences vaccinating behavior through individuals wanting to avoid health risks, and vaccinating behavior in turn influences disease incidence through herd immunity generated by vaccination [2]–[14].

A crucial assumption of these “behavior-incidence” models is that disease incidence feeds back on vaccinating behavior: a surge in disease incidence can convince individuals to start being vaccinated again. However, it is not immediately clear whether feedback is necessary to explain the time series of vaccine coverage in Figure 1: it may just reflect the gradual evolution of individuals' risk perception, irrespective of the influence of disease incidence.

In both vaccine scares, the publication of alleged vaccine risks was followed by a media firestorm in national newspapers, television, and radio [15], [16]. In light of this, the fact that it took 4–5 years for vaccine coverage to bottom out is puzzling. Peer opinion partly determines vaccine uptake [17], and social learning might explain the delay: to some extent, non-vaccinating behavior would have to spread from parent to parent.

For significant parts of many historical vaccine coverage time series, vaccine coverage is roughly constant if a vaccine scare is not occurring. It is relatively easy to make behavior-incidence models reproduce constant vaccine coverage because there are sufficient degrees of freedom in parameter space [7]. In contrast, vaccine scares constitute a more decisive test of these models, because a large part of the space of possible model dynamics is visited over the course of the vaccine scare, due to relatively rapid changes in vaccine coverage over time. Hence, we focus our analysis on the time periods during ongoing vaccine scares. Two vaccines scares in England & Wales offer ideal natural experiments for testing these models: the whole cell pertussis vaccine scare from the 1970s and the MMR vaccine scare from the 1990s. Our first objective was to determine whether a behavior-incidence model that includes social learning and disease incidence feedback can explain the vaccine coverage data from these two vaccine scares better than competing explanations that ignore social learning and/or feedback mechanisms. Our second objective was to determine whether this model could predict in advance the time evolution of vaccine coverage and disease incidence as observed in these two vaccine scares.

## Methods

We tested the behavior-incidence model in two stages. In stage one, we tested just the explanatory power of the behavioral component of the model on its own: we formulated a behavioral model based on a social learning process where vaccinating behavior depends on the disease incidence, and where disease incidence comes from the empirical data rather than being generated by a model. In stage two, we tested both the explanatory and predictive power of the full behavior-incidence model: we formulated a mathematical model of disease transmission and connected it to the behavioral model by making vaccinating behavior depend on disease incidence generated by the transmission model.

### Behavioral model

In stage one, we formulated a social learning process based on the imitation dynamic of evolutionary game theory [18]. An individual samples others in the population at a constant rate. If the sampled person is playing a different strategy and is receiving a higher payoff, the individual switches to that strategy with a probability proportional to the expected gain in payoff. The payoff gain depends on the difference between the penalty for being vaccinated and the penalty for risking infection. In our model, individuals can choose to vaccinate, or not to vaccinate (“vaccinator” versus “non-vaccinator” strategies). The infection penalty is the perceived probability of being infected—which we assumed is simply proportional to the current disease incidence—times the perceived cost of being infected. In stage one, we simply took disease incidence directly from the data in Figure 1 instead of an incidence model, resulting in a “behavioral model” rather than a full behavior-incidence model. The resulting equations for the behavioral model are
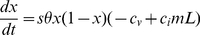
(1)where *x* is the proportion of vaccinators in the population at time *t*, *s* is the sampling rate, *θ* is the proportionality constant influencing the probability of switching strategies according to the expected gain in payoff, *c*_v_ is the penalty to vaccinate, *c*_i_ is the penalty for becoming infected, *L* is the number of case notifications at time *t* (taken from the data in Figure 1), and *m* is a proportionality constant governing the perceived probability of being infected (we note that for *m* and *θ* sufficiently small the relevant probabilities are always less than 1). The expression (−*c*_v_+*c*_i_ *mL*) is the payoff gain for switching strategies and its sign determines whether vaccinator or nonvaccinator is the favored switch. Equation (1) is derived in the Supporting Information (Text S1). Equation (1) can be further distilled to
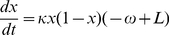
(2)where *κ* = *sθc_i_m* and ω = *c*_v_/*mc_i_*. This is the form of the model we use in the analysis. The parameter ω has absorbed *c*_v_ which, unlike other parameters, evolves over time as the perceived vaccination penalty changes during the vaccine scare.

### Risk evolution curves

We wanted to determine whether adding social learning and feedback in this way to some underlying description of how the perceived vaccination penalty evolves over time can better explain Figure 1. Hence, we formulated five risk evolution curves that govern how the perceived vaccination penalty could rise and fall during the scare. The function ω = ω(t) denotes the risk evolution curve describing time evolution of the vaccine penalty. ω(t) is constant at ω_pre_ until the vaccine scare, then climbs linearly for D_increase_ years to reach a maximum of σω_pre_ (where σ>1) and remains there for D_max_ years before declining linearly back to ω_pre_ over a period of D_decrease_ years. We explored five possible shapes for ω(t):

- Curve #1: instantaneous increase in perceived vaccine risk followed by linear decline: set D_increase_ = D_max_ = 0 and fit ω_pre_, σ, D_decrease_;
- Curve #2: instantaneous increase followed by plateau followed by instantaneous decline: set D_increase_ = D_decrease_ = 0 and fit ω_pre_, σ, D_max_;
- Curve #3: instantaneous increase followed by plateau followed by linear decline: set D_increase_ = 0 and fit ω_pre_, σ, D_decrease_, D_max_;
- Curve #4: linear increase followed by plateau followed by instantaneous decline: set D_decrease_ = 0 and fit ω_pre_, σ, D_increase_, D_max_;
- Curve #5: linear increase followed by plateau followed by linear decline: fit ω_pre_, σ, D_decrease_, D_increase_ , D_max_.

A diagram of ω(t) appears in Supporting Information (Figure S1).

These curves were not motivated by a specific mechanistic model of risk perception, but rather were intended to describe a wide range of possible functional forms requiring differing numbers of parameters, thus enabling the explanatory power of the behavioral model to be tested against a broad range of potential competing candidates, as opposed to a single candidate. Public health efforts to restore faith in a safe and efficacious vaccine are represented as the eventual decline in perceived vaccine risk in these risk evolution curves.

For each curve, we compared the parsimony (explanatory power) of the behavioral model with both social learning and feedback—Equation (2)—to three reduced behavioral models with: (a) social learning but no feedback:
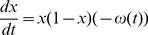
(3)(b) feedback but no social learning:
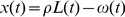
(4)where ρ is a proportionality constant, and (c) neither social learning nor feedback:
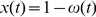
(5)These equations are derived in Supporting Information (Text S1).

We used the AICc—a modified Akaike Information Criterion [19]–[22]—to evaluate the parsimony of the four models under all five risk evolution curves for each model, yielding 20 candidates altogether. Information criteria have a strong rooting in information theory, and favor models that explain the data as well as possible with as few parameters as possible. The model with the most negative AICc score is the one with the greatest parsimony, suggesting that it is likely capturing crucial determinants of the observed dynamics. We obtained confidence intervals using a non-parametric bootstrapping method. Additional details on model fitting and bootstrapping appear in the Supporting Information (Text S1).

### Behavior-Incidence model

In stage 2, we evaluated the parsimony of the full behavior-incidence model. We augmented our behavioral model with a Susceptible-Infectious-Recovered (SIR) compartmental model that captures disease transmission processes. Despite their simplicity, similar models have been shown to capture pertussis and measles dynamics relatively well [23]–[25]. In the SIR model, individuals are either Susceptible, Infectious, or Recovered (immune). Susceptible individuals are infected at some rate and thereby moved to the Infectious compartment. From the Infectious compartment they recover at some rate and enter the Recovered compartment. Susceptible individuals who are efficaciously vaccinated are also moved to the Recovered compartment. Individuals are born into the Susceptible compartment at some rate, and leave the population due to death at some rate. For measles, the transmission rate was also made to vary seasonally [23]–[25]. For the behavioral component of the model, instead of making the perceived probability of being infected depend on the disease incidence data (*L*), it now depends on the disease prevalence generated by the SIR model (*I*). In turn, a proportion of infants are vaccinated according to the abundance of vaccinator strategists in the population at a given time (*x*), completing the feedback loop. The equations for the resulting behavior-incidence model are:
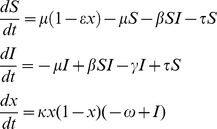
(6)where μ is the birth/death rate per capita, ε is the vaccine efficacy, β is the transmission rate, τ is the case importation rate, and γ is the recovery rate. For measles, a delay was also introduced between changes in incidence and changes in vaccine coverage, to capture phenomenologically the fact many parents have opted to delay immunization rather than avoid it altogether. As a result, for measles the *x* equation becomes
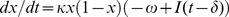
(7)where δ is the delay, in years. We opted to introduce a fixed delay in the equations rather than explicitly incorporate delayer strategies in order to keep the number of parameters relatively low.

The design of the parsimony analysis for the behavior-incidence model was similar to that of the behavioral model (see Supporting Information, Text S1). We note that the goodness of fit of the behavior-incidence model to disease incidence data does not, and cannot, contribute to the AICc score because in this respect there is no way to make a fair comparison between the behavior-incidence model (which is capable of predicting incidence) and the reduced models (two of which are not capable of predicting incidence, by definition).

In stage 2 we also tested the predictive power of the behavior-incidence model, under risk evolution curve #1. The slope of curve #1 is fixed at the start of the scare and does not change thereafter. This allowed us to fit the behavior-prevalence model under curve #1 to the early data points on both vaccine coverage and disease incidence in Figure 1 (*t*≤*t*_fit_), to see whether it can predict later data on vaccine coverage and disease incidence (*t*_fit_>*t*). We fitted disease incidence and vaccine coverage simultaneously, by minimizing a weighted sum of the residual sum of squares (RSS) for vaccine coverage and the RSS for disease incidence. We also conducted a probabilistic sensitivity analysis (PSA) to assess the sensitivity of these predictions to parameter uncertainty. PSA defines plausible intervals for crucial model parameters and initial conditions. For each model realization, samples are drawn from statistical distributions based on those intervals and the model is fitted using those parameter values. Over many such realizations it is possible to see how sensitive the model predictions are to variations in the input parameters. A bootstrapping analysis was also performed to further test model sensitivity to input parameter uncertainties and to acquire confidence intervals. Details of fitting, PSA and bootstrapping appear in the Supporting Information (Text S1).

## Results

### Stage one

**Figure 2:**
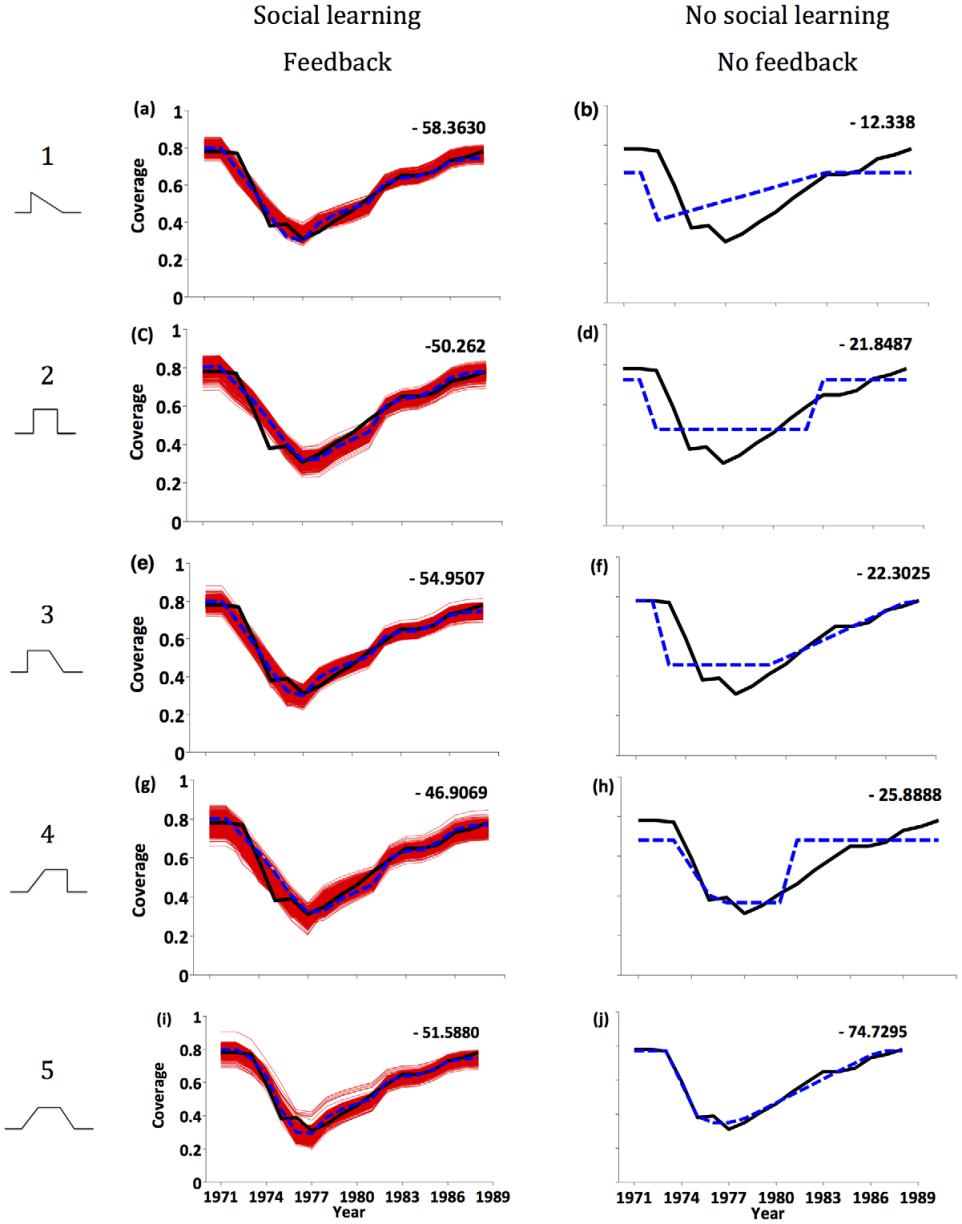
Parsimony analysis of behavioral model for the pertussis vaccine scare: vaccine coverage data (solid black line) and best-fitting model (dashed blue line) under behavioral model with both social learning and feedback (a, c, e, g, i) and reduced behavioral model with neither (b, d, f, h, j), under risk evolution curves #1–#5 (left-hand column). Red lines are 50 bootstrapped samples. Numerical values in subpanels are AICc scores: lower values indicate greater parsimony.

We analyzed both the whole cell pertussis vaccine scare and the MMR vaccine scare. For pertussis, the behavioral model with social learning and feedback fit the vaccine coverage data quite well under all risk evolution curves (Figure 2). In comparison, the two reduced models with feedback but no social learning, and social learning but no feedback, produced poor fits and were much less parsimonious (Supporting Information Figure S2). The third reduced model with neither social learning nor feedback also did worse in terms of fit and parsimony, except under risk evolution curve #5 (Figure 2). Thus, on the whole, adding social learning and feedback significantly improved model parsimony and fit. Results were very similar for MMR, with the behavioral model doing better in all cases except for the reduced model with neither social learning nor feedback under curve #5 (Supporting Information Figure S3). Confidence intervals and best-fitting parameter values appear in Supporting Information Tables S1, S2, S3, S4, S5. We discuss the significance of the reduced model with neither social learning nor feedback under curve #5 in the Discussion section.

### Stage two

**Figure 3:**
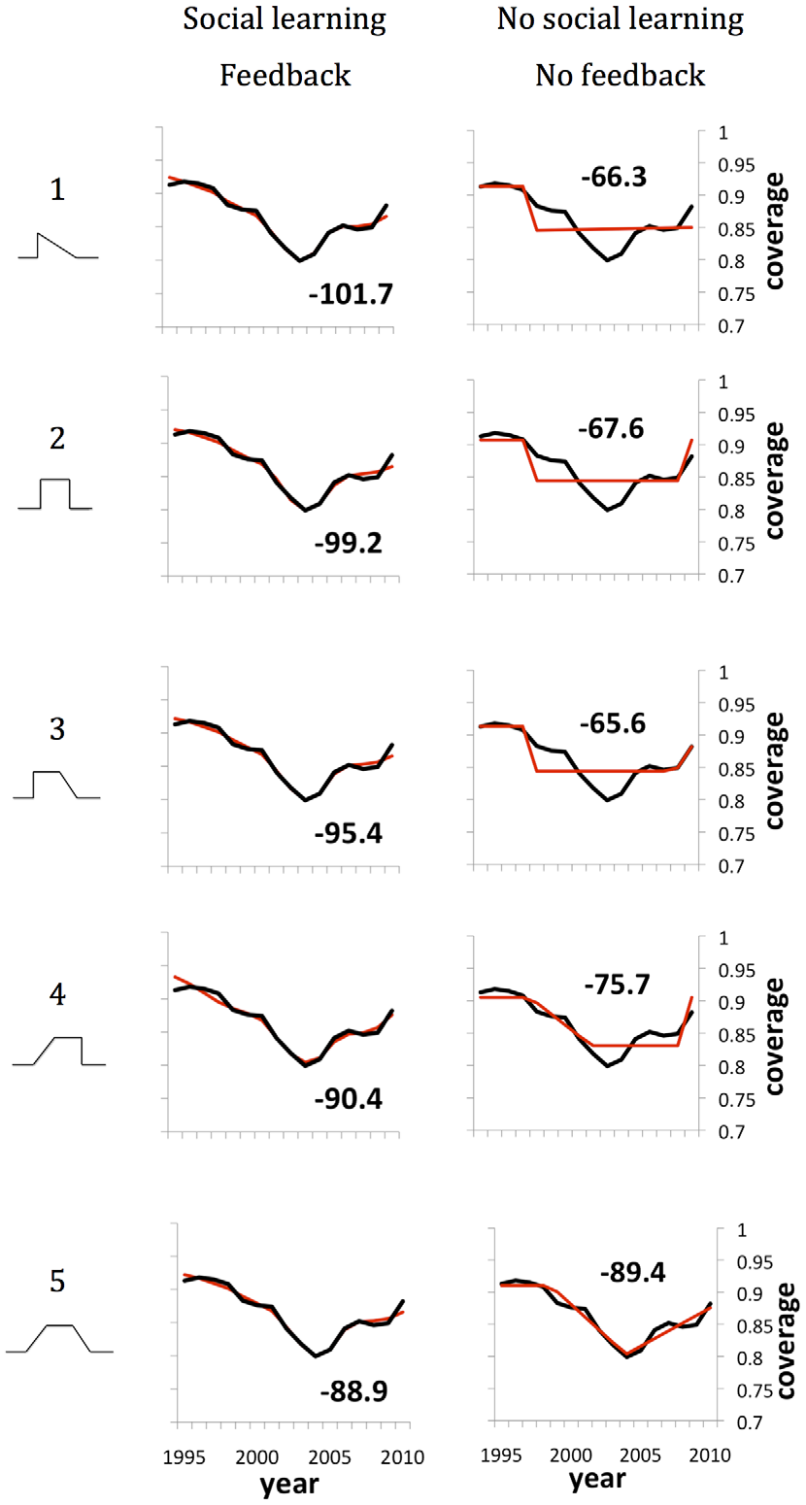
Parsimony analysis of behavior-incidence model and the reduced model with neither social learning nor feedback under risk evolution curves #1–#5 (left-hand column), for the MMR vaccine scare: best fitting model (red) and vaccine coverage data (black). The numerical value in the figure inset is the AICc score.

We repeated the parsimony analysis using the full behavior-incidence model, finding some further improvement in fit and parsimony relative to the three reduced behavioral models. For pertussis, the behavior-incidence model again achieves a better AICc score in all cases except for the model with neither social learning nor feedback under curve #5 (Supporting Information Figure S4). In contrast, for the case of MMR, the behavior-incidence model under curve #1 becomes the most parsimonious of all 20 candidates. Interestingly, its best-fitting solution for vaccine coverage is almost indistinguishable from the data for most of the vaccine scare (Figure 3; see Supporting Information Figures S5 for full results).

By comparing the fit of the behavior-incidence model to the fit of the reduced model with neither social learning nor feedback, under curve #1 for MMR (Figure 3), we can see the effects of adding social learning and feedback to an underlying model of risk perception evolution: social learning delays the trough in vaccine coverage (an effect that is also observed in the data, where vaccine coverage bottoms out many years after the hypothesized link between MMR vaccine and autism was published), and feedback allows the model to capture the undulations in vaccine coverage observed in the data, which appear to be caused by entrainment of vaccine coverage dynamics with disease dynamics.

The results for the reduced model with feedback but no social learning are also telling (Supporting Information Figure S4): although the overall trend in vaccine coverage is tracked approximately, the predicted vaccine coverage is too irregular because without the inertial effects of an imitation dynamic, vaccine coverage responds too rapidly to slight changes in infection prevalence and the coupled behavior-incidence dynamics become unstable.

In principle, a good fit could occur because the model is underdetermined: there are too many parameters for the amount of available data and thus the model is able to fit any arbitrary pattern by adjusting the parameter values appropriately. To rule out this possibility, we also fitted the model to randomly generated time series (correlated white noise) for the case of MMR. If the model were underdetermined, then the model should also be able to fit these arbitrary time series. In Supporting Information Figures S6, S7, S8, S9, S10, S11, S12, S13, S14, S15, S16, S17, we show that the model fits to these arbitrary time series are worse than its fit to the empirical vaccine coverage data in Figure 3, and thus the model may not be underdetermined.

**Figure 4:**
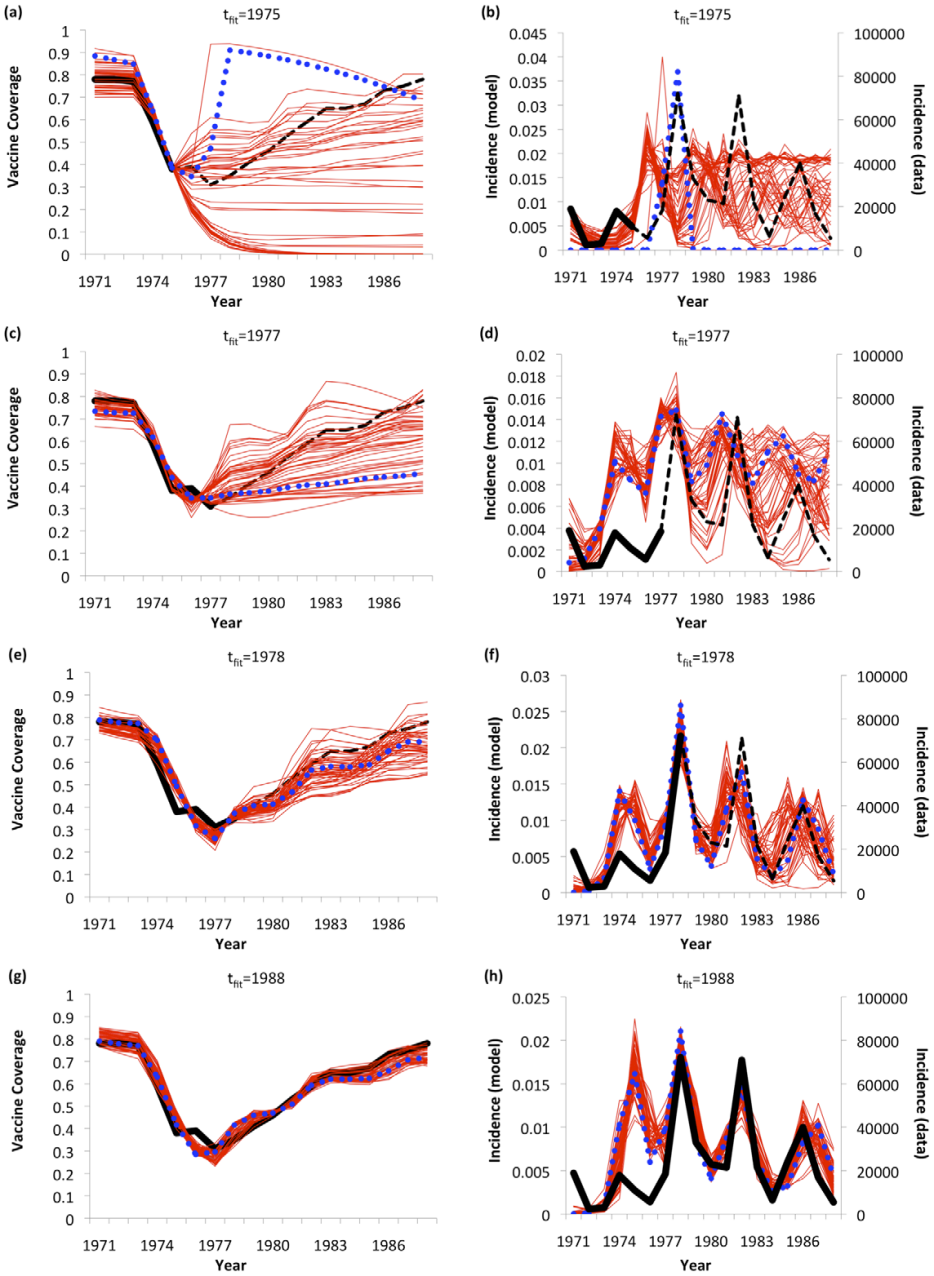
Predictive analysis of behavior-incidence model for the pertussis vaccine scare: predictions up until 1988 were made using data up until *t*_fit_ = 1975 (a, b); 1977 (c, d), 1978 (e, f) and 1988 (g, h) for both vaccine coverage (a, c, e, g) and case notifications (b, d, f, h). Best fitting model (blue dots), 50 realizations from PSA (red lines), vaccine coverage and disease incidence data used to fit model (*t*≤*t*_fit_; thick black lines), and data used to evaluate model predictions (*t*>*t*_fit_; dashed black lines) are shown.

In stage 2 we also evaluated the predictive power of the behavior-incidence model by fitting the model to the first part of vaccine coverage and disease incidence time series (*t*≤*t*_fit_) to see how well it predicts the second part (*t*>*t*_fit_). For pertussis, the model has little predictive power in the first few years of the scare: the best-fitting solution fails to capture the long-term dynamics of either vaccine coverage or disease dynamics, and the sampled realizations of the PSA are likewise inaccurate and widely scattered (*t*_fit_ = 1973; Figure 4a, b). This situation remains unchanged through 1977 (*t*_fit_ = 1977, Figure 4c, d). However, in 1978, the first large incidence peak occurs, resulting in an abrupt increase in predictive power: now, the best-fitting solution predicts both future vaccine coverage and disease dynamics fairly well up until 1988, and the sampled realizations of the PSA converge around future data points (*t*_fit_ = 1978; Figure 4e, f). Hence, the 1978 incidence peak acts to provide information that collapses model uncertainty, enabling reasonably accurate long-term predictions. This occurs despite the fact that—based on information available in 1978—it would not have been clear whether vaccine coverage had actually bottomed out or how quickly vaccine coverage would rebound.

The model also qualitatively captures the subtle undulations in vaccine coverage between 1982 and 1987 that are superimposed on the longer-term trend (Figure 4e): in both model and data, two incidence peaks occur during this time period, each followed shortly thereafter by a surge in vaccine coverage. However, the amplitude of the surges is larger in the model than in the data, and the first surge is predicted to occur a year before it actually happened. From the incidence plot (Figure 4f) we see that the model predicts the first incidence peak a year too soon, which is what causes the model to predict the first vaccine coverage a year too soon as well. This suggests that using a slightly more sophisticated transmission model might result in better alignment of predicted and observed vaccine coverage surges. These simulations highlight the fact that vaccine coverage in the data surges at exactly the time it should, if vaccine coverage were partly driven by disease dynamics. We also note that the ability of the model to track subtle undulations is responsible for much of the model's AICc score, especially in the case of MMR (Figure 3). From 1978 onward, model predictions are gradually refined and the vaccine coverage undulations become better aligned (Figure 4g, h; see Supporting Information Figure S18 for all *t*_fit_ values). However, even when *t*_fit_ = 1988 and the whole time series is used to fit the model, it continues to over-predict the magnitude of the first incidence peak; this may be partly explained by under-reporting of pertussis incidence in the early years of the vaccine scare when misdiagnosis would have been more likely. The model also places the first incidence peak in 1975, instead of 1974 when it actually occurred.

In the years preceding the time window shown in Figure 4, the modeled vaccine coverage is close to a steady state. The modeled vaccine coverage returns to this steady state after the scare is finished. This pattern is also observed in the vaccine coverage data. However, given that the whole cell pertussis vaccine was replaced with an acellular vaccine in the early 1990s, vaccine coverage data from this time period cannot be used to validate the model.

The results are qualitatively similar under the bootstrapping analysis: the bootstrapped predictions change abruptly in 1978, generating coherent and accurate predictions through 1988 (Supporting Information Figure S19). Using the whole time series to fit the model (*t*_fit_ = 1988), from the bootstrapping analysis we estimate that σ = 27 (95% CI: 19, 35), corresponding to a 27-fold increase in the perceived vaccine risk at the start of the vaccine scare. Other confidence intervals and best-fitting parameter values appear in Supporting Information Table S6.

**Figure 5:**
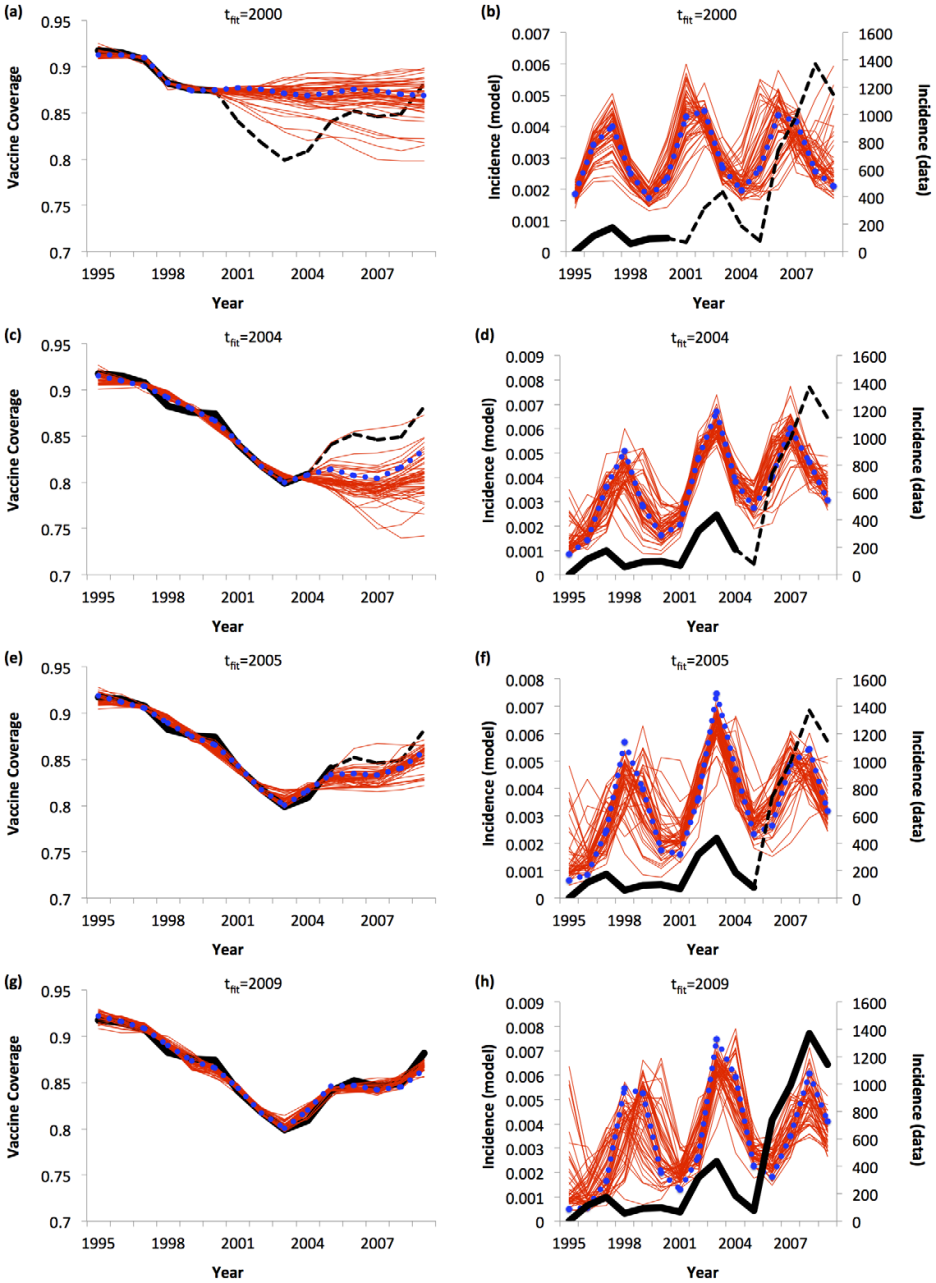
Predictive analysis of behavior-incidence model for the MMR vaccine scare: predictions up until 2009 were made using data up until *t*_fit_ = 2000 (a, b); 2004 (c, d), 2005 (e, f) and 2009 (g, h) for both vaccine coverage (a, c, e, g) and case notifications (b, d, f, h). Best fitting model (blue dots), 50 realizations from PSA (red lines), vaccine coverage and disease incidence data used to fit model (*t*≤*t*_fit_; thick black lines), and data used to evaluate model predictions (*t*>*t*_fit_; dashed black lines) are shown.

Predicting behavior-incidence dynamics during the MMR vaccine scare is more challenging. Vaccine coverage declined less. Measles did not become endemic until 2008 [1], so there is a lower volume of lab-confirmed cases with which to parameterize the model, and no large epidemic outbreaks until later. As a result, measles dynamics are highly stochastic until 2008, meaning that deterministic models such as the SIR model are less suited to describing this phase of the vaccine scare. Perhaps as a result of this, the model does not develop good predictive power until 2005 (Figure 5; see Supporting Information Figure S20 for all *t*_fit_ values). This appears to be stimulated by an unmistakable rebound of vaccine coverage, rather than by incidence peaks. Despite this limitation, by 2005, the model predicts vaccine coverage in 2009 relatively well. It also captures qualitatively the subtle undulations caused by feedback—the sudden deceleration of coverage in 2006–2007 and the subsequent acceleration in 2008–2009. Bootstrapping again yields similar results to PSA (Supporting Information Figure S21).

Using the whole time series to fit the model (*t*_fit_ = 2009), from the bootstrapping analysis we estimate that σ = 3.9 (95% CI: 3.1, 4.6), corresponding to a 4-fold increase in the perceived vaccine risk at the start of the vaccine scare. This value is much less than the 27-fold increase estimated for pertussis. For the delay δ, we estimate a biologically plausible value of 1.2 years (95% CI: 0.6, 1.8). The main effect of δ is to improve model fit by allowing peaks in the incidence data to stimulate correctly timed surges in the vaccine coverage data. If the delay is fixed at δ = 0, the alignment becomes worse. Other confidence intervals and best-fitting parameter values appear in Supporting Information Table S7.

In both vaccine scares, the fit to vaccine coverage is better than the fit to disease incidence data. This occurs because individuals weigh both infection risks and vaccine risks in their vaccinating decisions (Equation (6)), therefore vaccine coverage is determined both by disease incidence feedback and by the risk evolution curve. As a result, if the transmission model over-predicts incidence in some part of the time series, vaccine coverage can still be made to fit well by increasing the perception of vaccine risk during the same time period, such that an increase in the prevalence of infection is balanced by an increase in the perception of vaccine risk. For instance, in the first six years of the pertussis scare (where the model over-predicts the size of the incidence peak relative to subsequent incidence peaks), this can be accomplished by increasing the value of σ such that perceived risk jumps more significantly at the start of the vaccine scare. For risk evolution curve #1, this also elevates perceived vaccine risk later on in the time series, but not as much since vaccine risk tends to return to baseline over time and therefore the resulting incremental change in vaccine risk is smaller during the later years of the vaccine scare. Something similar can be said of MMR, which is why the timing of the incidence peaks appears to be more important for model fit than the relative size of incidence peaks.

## Discussion

Here we analyzed a relatively simple mathematical model of behavior-incidence dynamics. The model was based on evolutionary game theory, included both social learning and feedback of disease incidence on vaccinating behavior, and also included an exogenous description of how perceived vaccine risk evolves during a vaccine scare. We showed that the behavior-incidence model explains vaccine coverage data more parsimoniously than most reduced models with the same risk evolution curve but without social learning and/or feedback. More interestingly, in some circumstances, the behavior-incidence model can predict future vaccination coverage and disease incidence in a population where a vaccine scare has taken hold. These results suggest that strategic (game theoretical) interactions between individuals and social learning may be crucial governing mechanisms of the population response to a vaccine scare, in addition to changes in subjective vaccine risk perception.

The models with both social learning and feedback (both the behavioral model and the behavior-incidence model) were significantly more parsimonious than most other candidates. The exception was the reduced model with neither social learning nor feedback under curve #5, which did better in 3 of the 4 comparisons. In some sense, our experimental design “stacks the cards” against the behavior-incidence model: by adding a sufficient number of free parameters to the risk evolution curve it will always be possible to achieve an arbitrarily good AICc score without adding social learning or feedback (see Supporting Information, Text S1). At some point, enough parameters are added to allow a “naked” risk evolution curve to outperform the corresponding behavior-incidence model; in the current analysis that point was reached with curve #5 with its five free parameters. However, our risk evolution curves were intended to represent phenomenologically a broad range of potential competing models, and in practice it may not even be possible to construct a mechanistic risk evolution model that can track the data as closely as curve #5 does. For example, we note that a simple SIR-type rumor propagation model could not replicate the approximately linear decline and recovery in vaccine coverage seen in the case of pertussis.

Considering these issues, it may not be appropriate to interpret our results in terms of a classical model selection exercise (where the model with the best AICc score is adopted). Additionally, we have little idea of how perceived vaccine risk actually evolved during these vaccine scares and hence it is difficult to construct a mechanistic risk evolution model in the first place, which makes a true model comparison elusive. Because of the apparent difficulties in teasing out the effects of the inherent dynamics of a vaccine scare from those of social learning and feedback, we refrain from interpreting our results as a classical model selection exercise. Rather, we choose to emphasize that a theoretically motivated approach consistent with human behavior improves model fit with little or no parsimony penalty, even when the underlying risk evolution curve is very crude (such as curves #1–#4).

Adding layers of sophistication to the model by including serious outcomes, combination versus single vaccines, age structure, spatial structure, or stochasticity may further improve the model's predictive power. These aspects represent opportunities for future work. Likewise, introducing a mechanistic model of how risk perception evolves instead of imposing risk evolution curves is worth pursuing, particularly in light of the interpretation caveats described in the previous paragraph. For example, this could take the form of a more mechanistic description of the impact of public health efforts such as information campaigns. However, the parsimony and predictive power of the model even without these extensions is considerable, and may be attributable to tight coupling between vaccinating behavior and disease incidence.

This research illustrates the importance of choosing the right transmission model when constructing a behavior-incidence model. Whooping cough incidence during the whole cell pertussis vaccine scare entered the regime of deterministic dynamics (widespread and unbroken chains of transmission), meaning that a simple, deterministic SIR model could capture the incidence peaks relatively well. However, measles incidence during the MMR scare was in a highly stochastic regime for most of the vaccine scare, which may explain the worse fit of the deterministic SIR model in that case.

A significant model limitation is the necessity to choose a weight governing how much the overall goodness of fit is determined by model fit to vaccine coverage versus the model fit to disease incidence. In the case of MMR, the fit to disease incidence was not weighted very strongly, on account of the poor ability of the deterministic model to fit stochastic disease dynamics. When model fit to both incidence and vaccine coverage is good, then the choice of *w* should not matter. Otherwise, knowing which value of *w* to choose requires experimentation with the data and therefore forces use of large values of *t*_fit_, which means the predictive capacity of the model is less.

Another model limitation is that, in the predictive analysis, the behavioral model is ‘trained’ on modeled incidence for *t*<*t*_fit_, rather than on actual incidence. This amounts to assuming that individuals were making vaccinating decisions based on modeled incidence, rather than on the incidence dynamics that the population actually experienced. One way to avoid this would be to fit the behavioral model to historical incidence data (*t*≤*t*_fit_) and then rely on modeled incidence data for projections into the future (*t*>*t*_fit_). However, there are technical difficulties arising from the switch at *t* = *t*_fit_ that would make this approach problematic. In particular, because of under-reporting in the empirical data, it would be easy to ‘confuse’ the behavioral model by switching its dependence from empirical incidence data to modeled incidence data at *t* = *t*_fit_. Moreover this model limitation is not a problem if agreement between modeled and empirical incidence is sufficiently close. Hence, ideally, it is better to train the behavioral model on modeled incidence for *t*<*t*_fit_. In any case, the issue of how to design good tests of the predictive ability of behavior-incidence models requires more thought.

The model cannot predict when a vaccine scare will occur since this presumably depends on singular historical events, such as publication of a study linking a vaccine to health risks. The model also requires data from the first years of a vaccine scare to predict subsequent years. In our analysis, we fitted the parameter σ that determines how much the vaccine penalty jumps when the scare starts. The predictive power of the model could increase if σ were known from the start. This is possible in principle, since it could be estimated from population surveys after a vaccine scare begins. This also represents opportunity for future work.

In 2003, polio was on the verge of global eradication when a vaccine scare in northern Nigeria caused an international resurgence of the disease [26]. Our results suggest that vaccine scares or other forms of “free riding” could become more common as eradication goals are approached for more vaccine-preventable diseases. Behavior-incidence models may help mitigate the impact of vaccine scares, and assist in planning the global eradication endgame against some infectious diseases.

## Supporting Information

Figure S1 Schematic diagram of risk evolution curves.
(PDF)

Figure S2 Parsimony analysis of the four behavioral models (horizontal dimension) under five evolution curves (vertical dimension) for pertussis vaccine scare. Solid black line is whole cell pertussis vaccine coverage. Dashed blue line is best fit of model to data. Red lines are bootstrapped fits. Numerical values in inset are AICc values of the best-fitting model.
(PDF)

Figure S3 Parsimony analysis of the four behavioral models (horizontal dimension) under five evolution curves (vertical dimension) for MMR vaccine scare. Solid black line is MMR vaccine coverage. Dashed blue line is best fit of model to data. Red lines are bootstrapped fits. Numerical values in inset are AICc values of the best-fitting model.
(PDF)

Figure S4 Parsimony analysis of behavior-incidence model, pertussis vaccine scare. Best fitting model (red) versus data (black) on whole cell pertussis vaccine uptake, for 5 risk evolution curves and 4 cases, using the behavior-incidence model. The numerical value in the inset of each subpanel is the corresponding AICc value for the fit. See page 2 for definition of risk evolution curves.
(PDF)

Figure S5 Parsimony analysis of behavior-incidence model, MMR vaccine scare. Best fitting model (red) versus data (black) on MMR vaccine uptake, for 5 risk evolution curves and 4 cases, using the behavior-incidence model. The numerical value in the inset of each subpanel is the corresponding AICc value for the fit. See page 2 for definition of risk evolution curves.
(PDF)

Figure S6 Best fit of behaviour model (red) to MMR vaccine coverage data and 10 sets of correlated white noise data and (black), for risk evolution curve #1. Also shown are goodness-of-fit and AICc of best fit (figure inset). Vertical scales range from 0.7 to 1.0; horizontal from 1995 to 2009.
(PDF)

Figure S7 Best fit of behaviour model (red) to MMR vaccine coverage data and 10 sets of correlated white noise data and (black), for risk evolution curve #2. Also shown are goodness-of-fit and AICc of best fit (figure inset). Vertical scales range from 0.7 to 1.0; horizontal from 1995 to 2009.
(PDF)

Figure S8 Best fit of behaviour model (red) to MMR vaccine coverage data and 10 sets of correlated white noise data and (black), for risk evolution curve #3. Also shown are goodness-of-fit and AICc of best fit (figure inset). Vertical scales range from 0.7 to 1.0; horizontal from 1995 to 2009.
(PDF)

Figure S9 Best fit of behaviour model (red) to MMR vaccine coverage data and 10 sets of correlated white noise data and (black), for risk evolution curve #4. Also shown are goodness-of-fit and AICc of best fit (figure inset). Vertical scales range from 0.7 to 1.0; horizontal from 1995 to 2009.
(PDF)

Figure S10 Best fit of behaviour model (red) to MMR vaccine coverage data and 10 sets of correlated white noise data and (black), for risk evolution curve #5. Also shown are goodness-of-fit and AICc of best fit (figure inset). Vertical scales range from 0.7 to 1.0; horizontal from 1995 to 2009.
(PDF)

Figure S11 Best fit of behaviour model (red) to MMR vaccine coverage data and 10 sets of correlated white noise data and (black), for risk evolution curve #6. Also shown are goodness-of-fit and AICc of best fit (figure inset). Vertical scales range from 0.7 to 1.0; horizontal from 1995 to 2009. Model was not fitted to vaccine coverage data using this risk evolution curve since a constant perceived vaccine penalty (curve #6) would correspond to no vaccine scare having occurred.
(PDF)

Figure S12 Best fit of behaviour- incidence model (red) to MMR vaccine coverage data and 10 sets of correlated white noise data and (black), for risk evolution curve #1. Also shown are log of maximum likelihood function and AICc of best fit (figure inset). Vertical scales range from 0.7 to 1.0; horizontal from 1995 to 2009.
(PDF)

Figure S13 Best fit of behaviour- incidence model (red) to MMR vaccine coverage data and 10 sets of correlated white noise data and (black), for risk evolution curve #2. Also shown are log of maximum likelihood function and AICc of best fit (figure inset). Vertical scales range from 0.7 to 1.0; horizontal from 1995 to 2009.
(PDF)

Figure S14 Best fit of behaviour- incidence model (red) to MMR vaccine coverage data and 10 sets of correlated white noise data and (black), for risk evolution curve #3. Also shown are log of maximum likelihood function and AICc of best fit (figure inset). Vertical scales range from 0.7 to 1.0; horizontal from 1995 to 2009.
(PDF)

Figure S15 Best fit of behaviour- incidence model (red) to MMR vaccine coverage data and 10 sets of correlated white noise data and (black), for risk evolution curve #4. Also shown are log of maximum likelihood function and AICc of best fit (figure inset). Vertical scales range from 0.7 to 1.0; horizontal from 1995 to 2009.
(PDF)

Figure S16 Best fit of behaviour- incidence model (red) to MMR vaccine coverage data and 10 sets of correlated white noise data and (black), for risk evolution curve #5. Also shown are log of maximum likelihood function and AICc of best fit (figure inset). Vertical scales range from 0.7 to 1.0; horizontal from 1995 to 2009.
(PDF)

Figure S17 Best fit of behaviour-incidence model (red) to 10 sets of correlated white noise data and (black), for risk evolution curve #6 Also shown are log of maximum likelihood function and AICc of best fit (figure inset). Vertical scales range from 0.7 to 1.0; horizontal 1995 to 2009. Model was not fitted to vaccine coverage data using this risk evolution curve since a constant perceived vaccine penalty (curve #6) would correspond to no vaccine scare having occurred.
(PDF)

Figure S18 PSA Results, Pertussis, *t*_fit_ from 1975 to 1988. Solid black line represents vaccine coverage/incidence data for *t≤t*_fit_; dashed black line represents vaccine coverage/incidence data for *t>t*_fit_ (data from years *t≤t*_fit_ were used to fit model and produce model extrapolation to *t*>*t*_fit_); dotted blue line represents the best fit of model to data for given value of *t*_fit_; thin red lines represent 50 Monte Carlo samples for a given value of *t*_fit_.
(PDF)

Figure S19 Bootstrapping Results for Pertussis, *t*_fit_ from 1975 to 1988. Solid black line represents vaccine coverage/incidence data for *t≤t*_fit_; dashed black line represents vaccine coverage/incidence data for *t>t*_fit_ (data from years *t≤t*_fit_ were used to fit model and produce model extrapolation to *t*>*t*_fit_); dotted blue line represents the best fit of model to data for given value of *t*_fit_; thin red lines represent 50 bootstrap samples for a given value of *t*_fit_.
(PDF)

Figure S20 PSA for MMR, *t*_fit_ values from 1997 to 2009. Solid black line represents vaccine coverage/incidence data for *t≤t*_fit_; dashed black line represents vaccine coverage/incidence data for *t>t*_fit_ (data from years *t≤t*_fit_ were used to fit model and produce model extrapolation to *t*>*t*_fit_); dotted blue line represents the best fit of model to data for given value of *t*_fit_; thin red lines represent 50 Monte Carlo samples for a given value of *t*_fit_.
(PDF)

Figure S21 Bootstrapping Results for MMR, *t*_fit_ from 1997 to 2009. Solid black line represents vaccine coverage/incidence data for *t≤t*_fit_; dashed black line represents vaccine coverage/incidence data for *t>t*_fit_ (data from years *t≤t*_fit_ were used to fit model and produce model extrapolation to *t*>*t*_fit_); dotted blue line represents the best fit of model to data for given value of *t*_fit_; thin red lines represent 50 bootstrap samples for a given value of *t*_fit_.
(PDF)

Table S1 Confidence interval of fitted parameters for all 5 risk evolution curves models for the behavioral model with social learning and feedback, derived from bootstrapping.
(PDF)

Table S2 Fitting results for behavioral model with social learning and feedback under 5 risk evolution curves.
(PDF)

Table S3 Fitting results for behavioral model with social learning but no feedback under 5 risk evolution curves.
(PDF)

Table S4 Fitting results for behavioral model with feedback but no social learning under 5 risk evolution curves.
(PDF)

Table S5 Fitting results for behavioral model with no feedback and no social learning under 5 risk evolution curves.
(PDF)

Table S6 Estimated parameter values from bootstrapping for behavior-incidence model for Pertussis. Values represent median (median −2 standard deviations, median +2 standard deviations) from 50 bootstrap samples.
(PDF)

Table S7 Estimated parameter values from bootstrapping for behavior-incidence model for MMR. Values represent median (median −2 standard deviations, median +2 standard deviations) from 50 bootstrap samples.
(PDF)

Text S1 Methods.
(PDF)
