# Supplementary figures and images for: Evolutionary Game Theory and Social Learning Can Determine How Vaccine Scares Unfold
Source: PLoS Comput Biol. 2012 Apr 5;8(4):e1002452. doi: 10.1371/journal.pcbi.1002452 (PMC3320575; doi:10.1371/journal.pcbi.1002452)

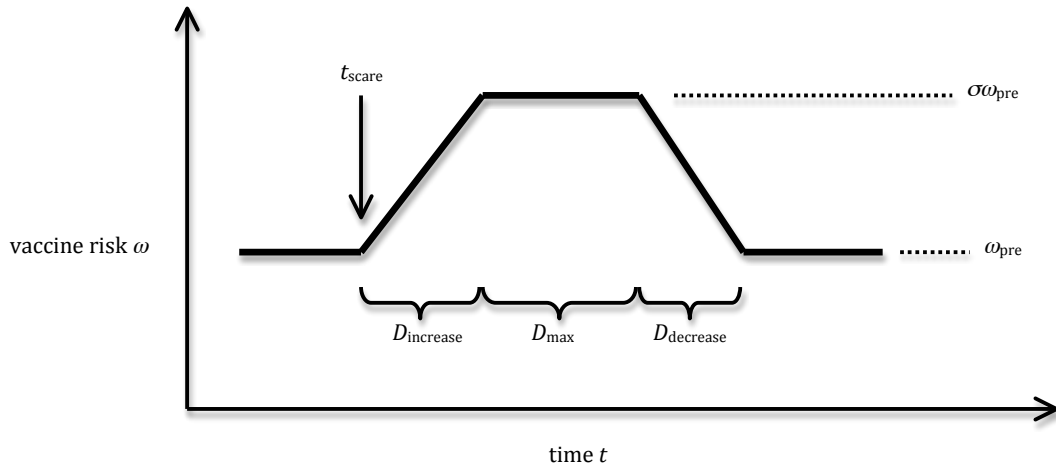

Supplement: Figure S1 — Schematic diagram of risk evolution curves. (PDF) [file pcbi.1002452.s001.pdf]

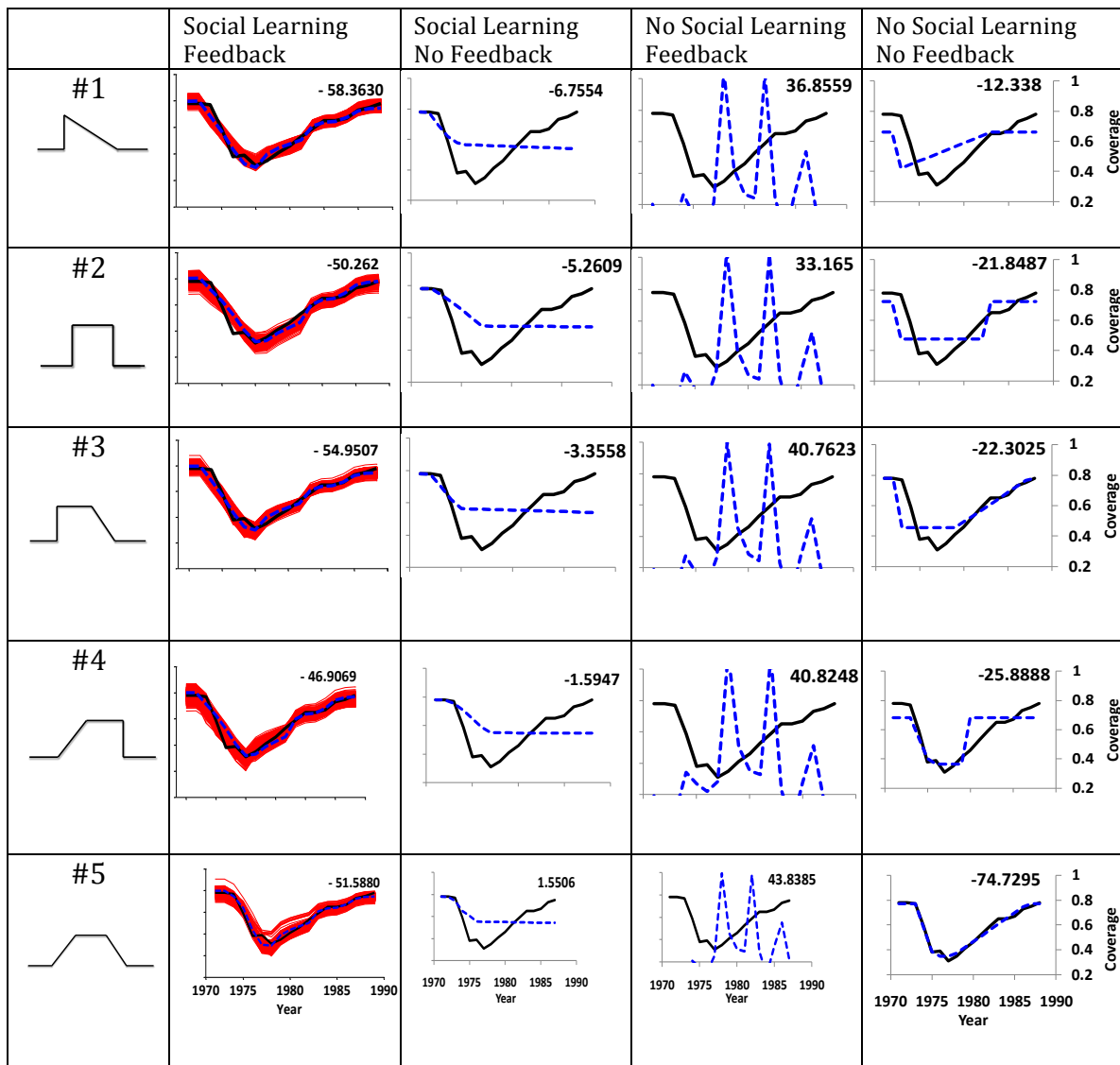

Supplement: Figure S2 — Parsimony analysis of the four behavioral models (horizontal dimension) under five evolution curves (vertical dimension) for pertussis vaccine scare. Solid black line is whole cell pertussis vaccine coverage. Dashed blue line is best fit of model to data. Red lines are bootstrapped fits. Numerical values in inset are AICc values of the best-fitting model. (PDF) [file pcbi.1002452.s002.pdf]

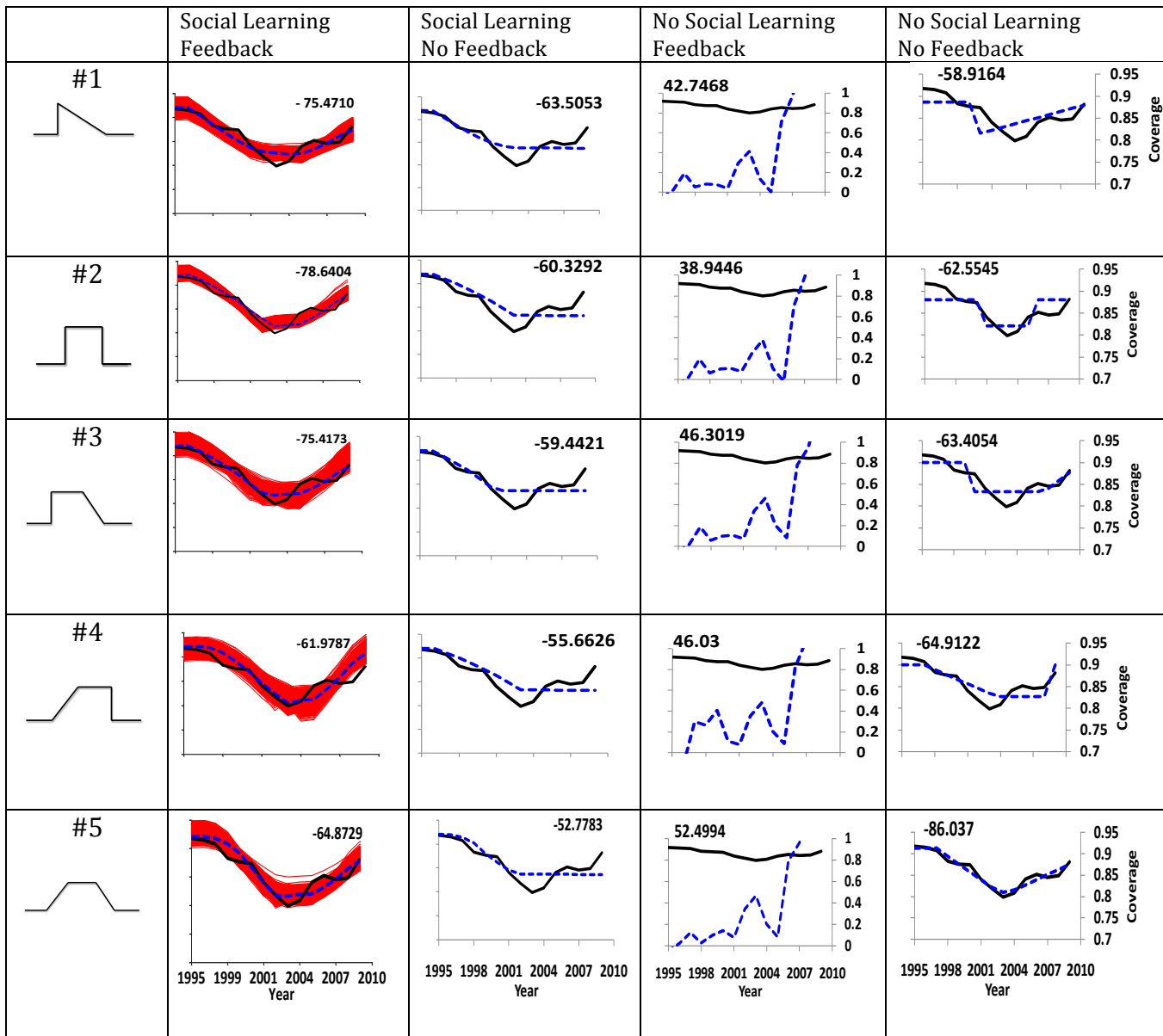

Supplement: Figure S3 — Parsimony analysis of the four behavioral models (horizontal dimension) under five evolution curves (vertical dimension) for MMR vaccine scare. Solid black line is MMR vaccine coverage. Dashed blue line is best fit of model to data. Red lines are bootstrapped fits. Numerical values in inset are AICc values of the best-fitting model. (PDF) [file pcbi.1002452.s003.pdf]

No social learning

No feedback

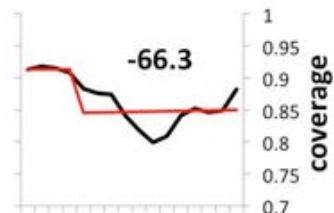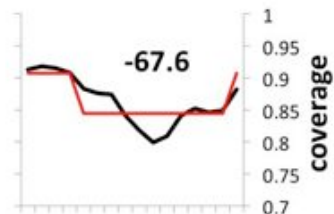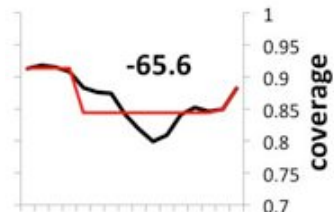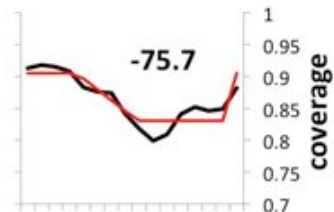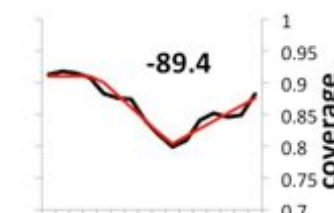

1995 2000 2005 2010  
year

Supplement: Figure S5 — Parsimony analysis of behavior-incidence model, MMR vaccine scare. Best fitting model (red) versus data (black) on MMR vaccine uptake, for 5 risk evolution curves and 4 cases, using the behavior-incidence model. The numerical value in the inset of each subpanel is the corresponding AICc value for the fit. See page 2 for definition of risk evolution curves. (PDF) [file pcbi.1002452.s005.pdf]

## Best fit of model to MMR vaccine coverage data

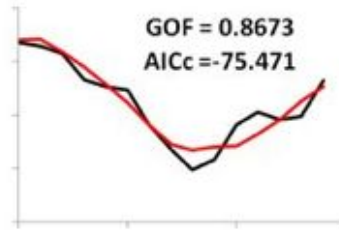

## Best fit of model to correlated white noise data

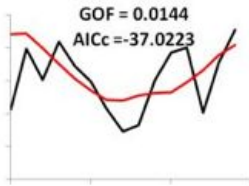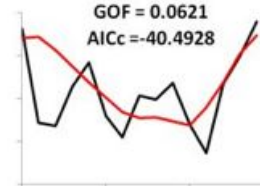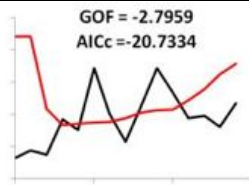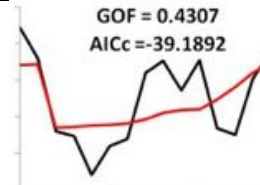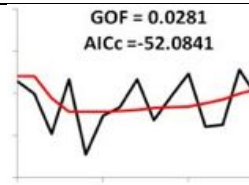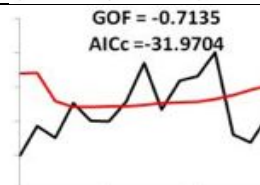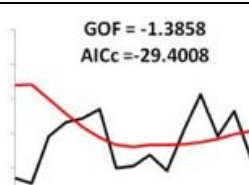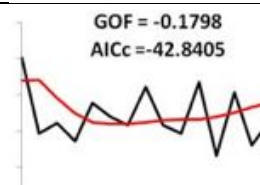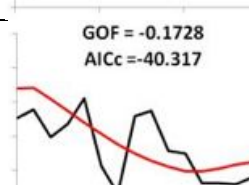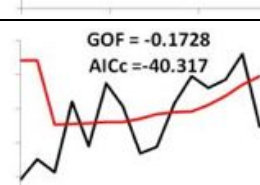

Supplement: Figure S6 — Best fit of behaviour model (red) to MMR vaccine coverage data and 10 sets of correlated white noise data and (black), for risk evolution curve #1. Also shown are goodness-of-fit and AICc of best fit (figure inset). Vertical scales range from 0.7 to 1.0; horizontal from 1995 to 2009. (PDF) [file pcbi.1002452.s006.pdf]

## Best fit of model to MMR vaccine coverage data

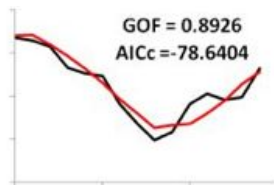

## Best fit of model to correlated white noise data

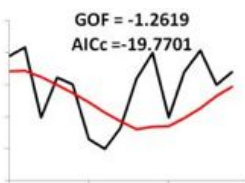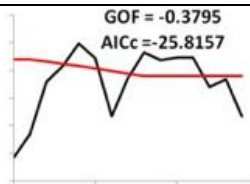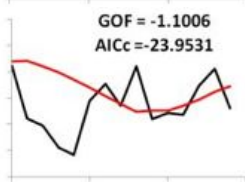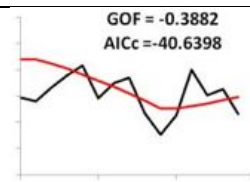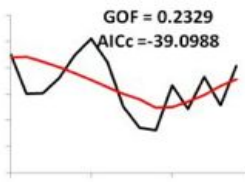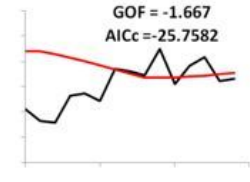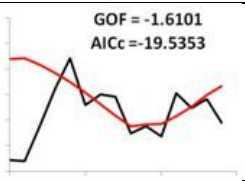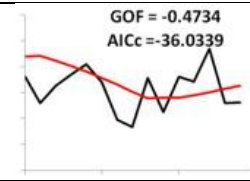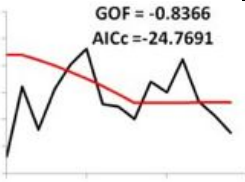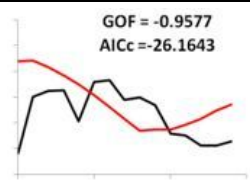

Supplement: Figure S7 — Best fit of behaviour model (red) to MMR vaccine coverage data and 10 sets of correlated white noise data and (black), for risk evolution curve #2. Also shown are goodness-of-fit and AICc of best fit (figure inset). Vertical scales range from 0.7 to 1.0; horizontal from 1995 to 2009. (PDF) [file pcbi.1002452.s007.pdf]

## Best fit of model to MMR vaccine coverage data

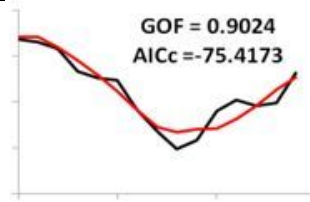

## Best fit of model to correlated white noise data

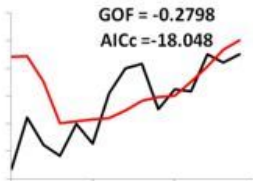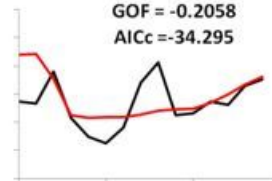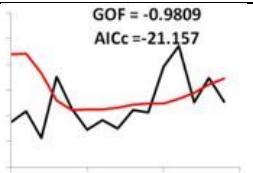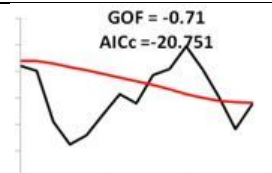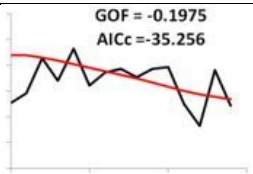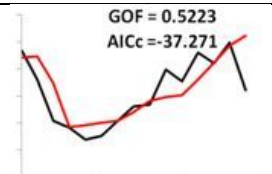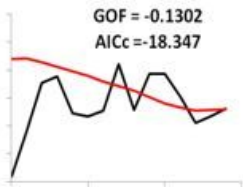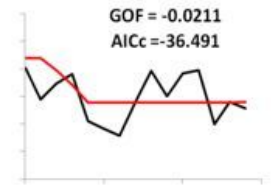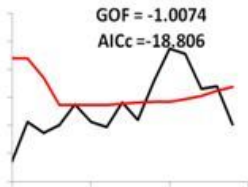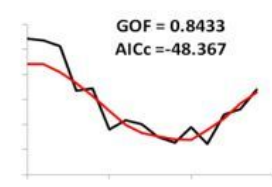

Supplement: Figure S8 — Best fit of behaviour model (red) to MMR vaccine coverage data and 10 sets of correlated white noise data and (black), for risk evolution curve #3. Also shown are goodness-of-fit and AICc of best fit (figure inset). Vertical scales range from 0.7 to 1.0; horizontal from 1995 to 2009. (PDF) [file pcbi.1002452.s008.pdf]

## Best fit of model to MMR vaccine coverage data

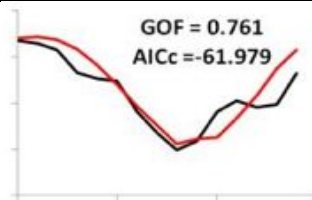

## Best fit of model to correlated white noise data

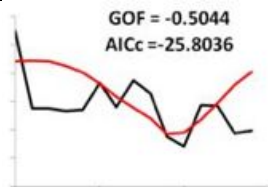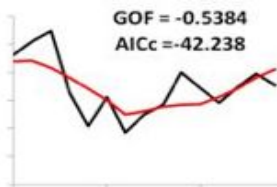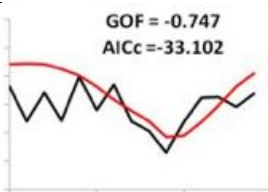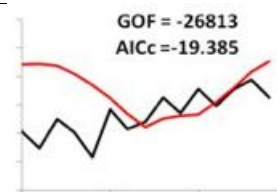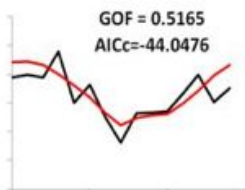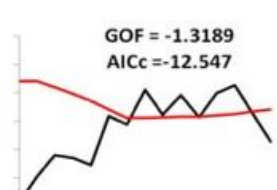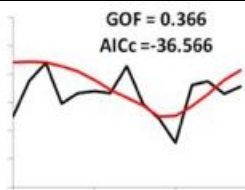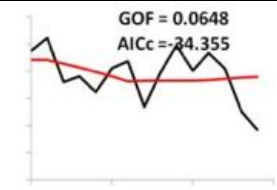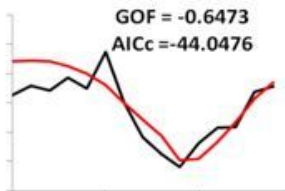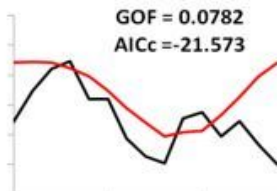

Supplement: Figure S9 — Best fit of behaviour model (red) to MMR vaccine coverage data and 10 sets of correlated white noise data and (black), for risk evolution curve #4. Also shown are goodness-of-fit and AICc of best fit (figure inset). Vertical scales range from 0.7 to 1.0; horizontal from 1995 to 2009. (PDF) [file pcbi.1002452.s009.pdf]

## Best fit of model to MMR vaccine coverage data

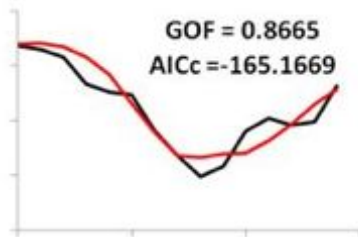

## Best fit of model to correlated white noise data

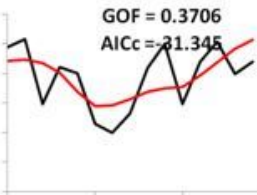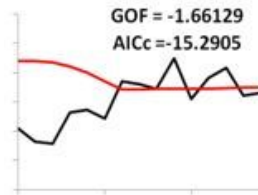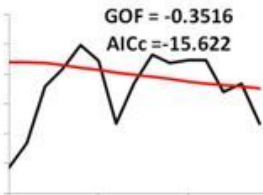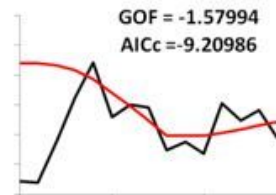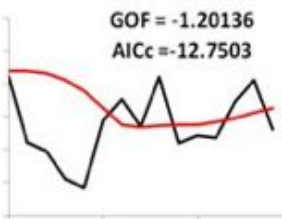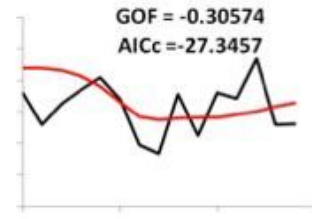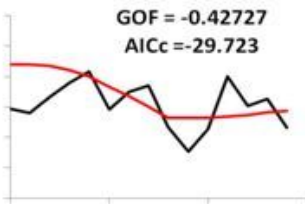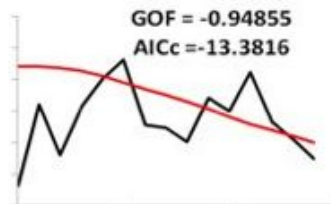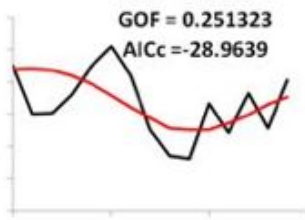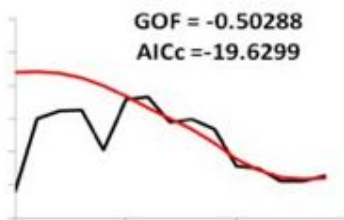

Supplement: Figure S10 — Best fit of behaviour model (red) to MMR vaccine coverage data and 10 sets of correlated white noise data and (black), for risk evolution curve #5. Also shown are goodness-of-fit and AICc of best fit (figure inset). Vertical scales range from 0.7 to 1.0; horizontal from 1995 to 2009. (PDF) [file pcbi.1002452.s010.pdf]

# Best fit of model to MMR vaccine coverage data

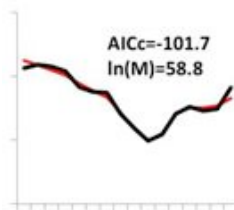

## Best fit of model to correlated white noise data

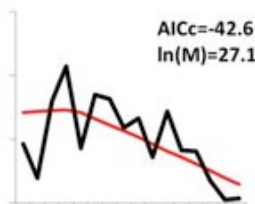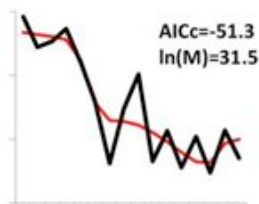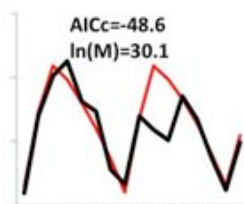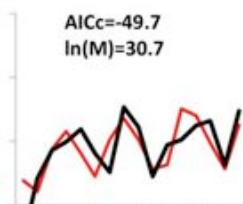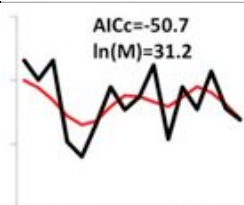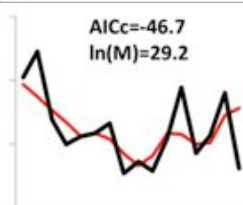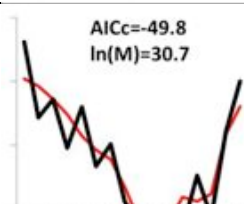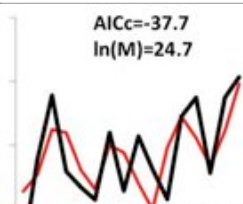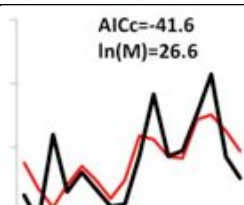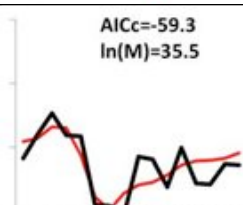

Supplement: Figure S12 — Best fit of behaviour- incidence model (red) to MMR vaccine coverage data and 10 sets of correlated white noise data and (black), for risk evolution curve #1. Also shown are log of maximum likelihood function and AICc of best fit (figure inset). Vertical scales range from 0.7 to 1.0; horizontal from 1995 to 2009. (PDF) [file pcbi.1002452.s012.pdf]

# Best fit of model to MMR vaccine coverage data

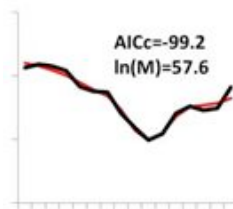

## Best fit of model to correlated white noise data

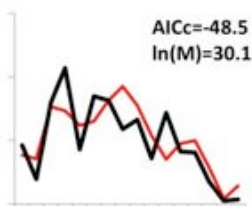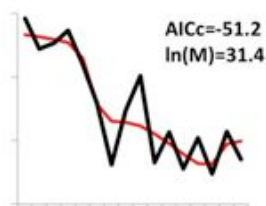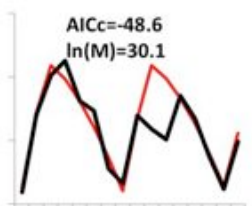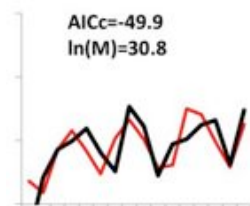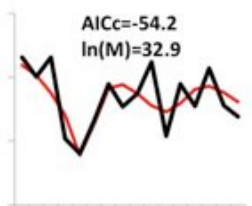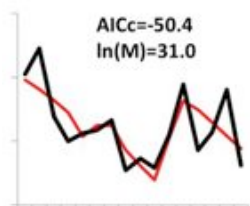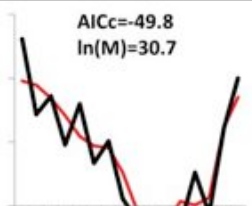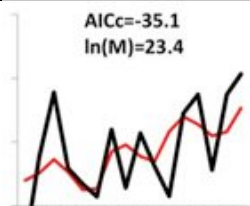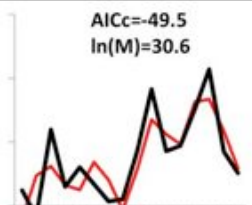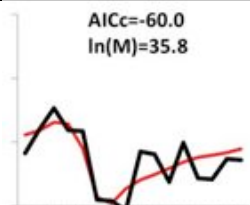

Supplement: Figure S13 — Best fit of behaviour- incidence model (red) to MMR vaccine coverage data and 10 sets of correlated white noise data and (black), for risk evolution curve #2. Also shown are log of maximum likelihood function and AICc of best fit (figure inset). Vertical scales range from 0.7 to 1.0; horizontal from 1995 to 2009. (PDF) [file pcbi.1002452.s013.pdf]

# Best fit of model to MMR vaccine coverage data

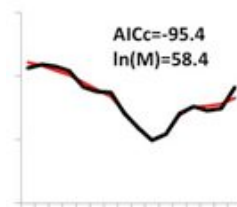

## Best fit of model to correlated white noise data

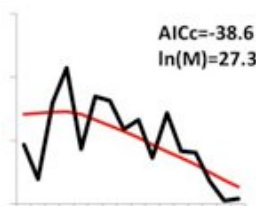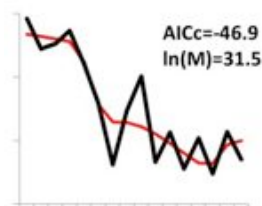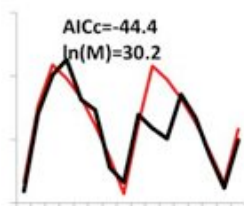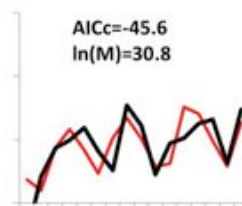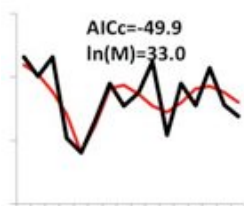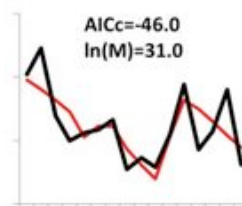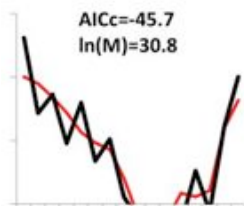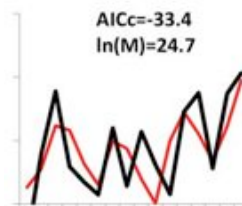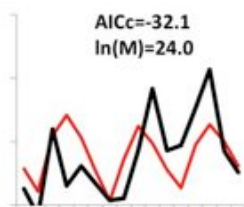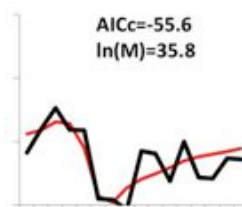

Supplement: Figure S14 — Best fit of behaviour- incidence model (red) to MMR vaccine coverage data and 10 sets of correlated white noise data and (black), for risk evolution curve #3. Also shown are log of maximum likelihood function and AICc of best fit (figure inset). Vertical scales range from 0.7 to 1.0; horizontal from 1995 to 2009. (PDF) [file pcbi.1002452.s014.pdf]

# Best fit of model to MMR vaccine coverage data

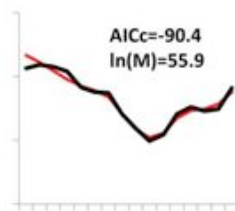

## Best fit of model to correlated white noise data

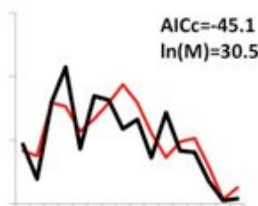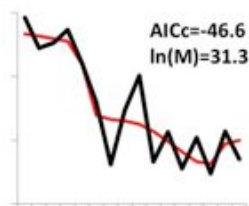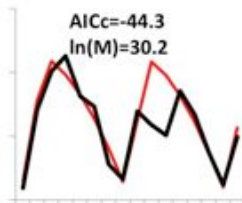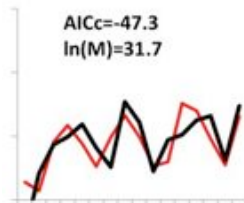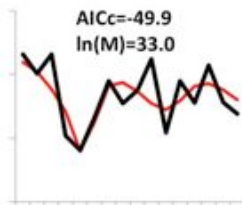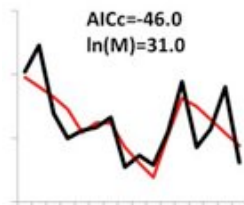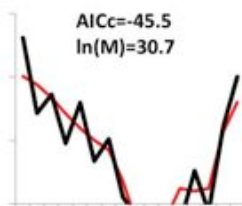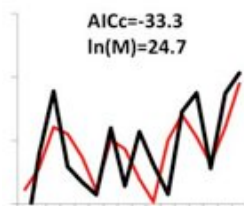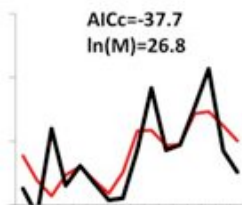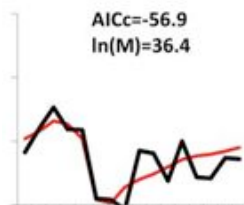

Supplement: Figure S15 — Best fit of behaviour- incidence model (red) to MMR vaccine coverage data and 10 sets of correlated white noise data and (black), for risk evolution curve #4. Also shown are log of maximum likelihood function and AICc of best fit (figure inset). Vertical scales range from 0.7 to 1.0; horizontal from 1995 to 2009. (PDF) [file pcbi.1002452.s015.pdf]

# Best fit of model to MMR vaccine coverage data

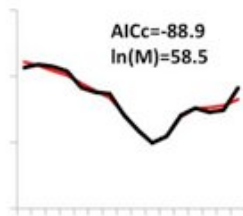

## Best fit of model to correlated white noise data

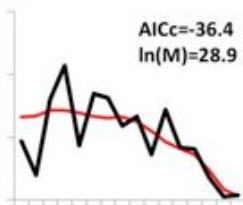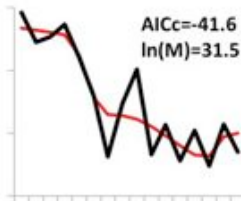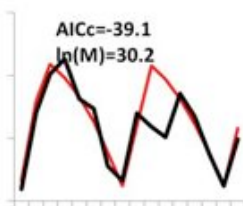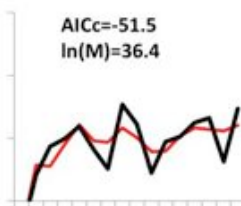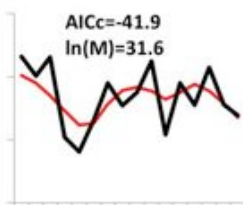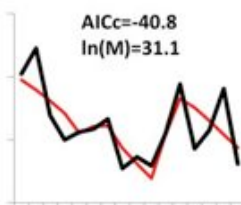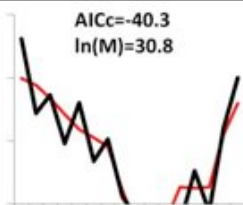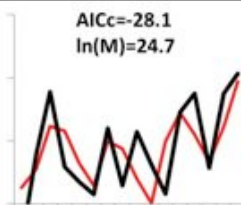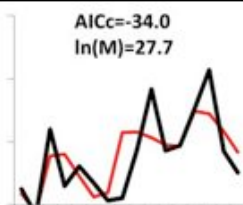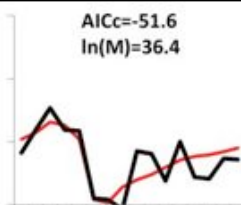

Supplement: Figure S16 — Best fit of behaviour- incidence model (red) to MMR vaccine coverage data and 10 sets of correlated white noise data and (black), for risk evolution curve #5. Also shown are log of maximum likelihood function and AICc of best fit (figure inset). Vertical scales range from 0.7 to 1.0; horizontal from 1995 to 2009. (PDF) [file pcbi.1002452.s016.pdf]
