# Supplementary material for: Evolutionary Game Theory and Social Learning Can Determine How Vaccine Scares Unfold
Source: PLoS Comput Biol. 2012 Apr 5;8(4):e1002452. doi: 10.1371/journal.pcbi.1002452 (PMC3320575; doi:10.1371/journal.pcbi.1002452)
Supplement: Figure S4 — Parsimony analysis of behavior-incidence model, pertussis vaccine scare. Best fitting model (red) versus data (black) on whole cell pertussis vaccine uptake, for 5 risk evolution curves and 4 cases, using the behavior-incidence model. The numerical value in the inset of each subpanel is the corresponding AICc value for the fit. See page 2 for definition of risk evolution curves. (PDF) [file pcbi.1002452.s004.pdf]

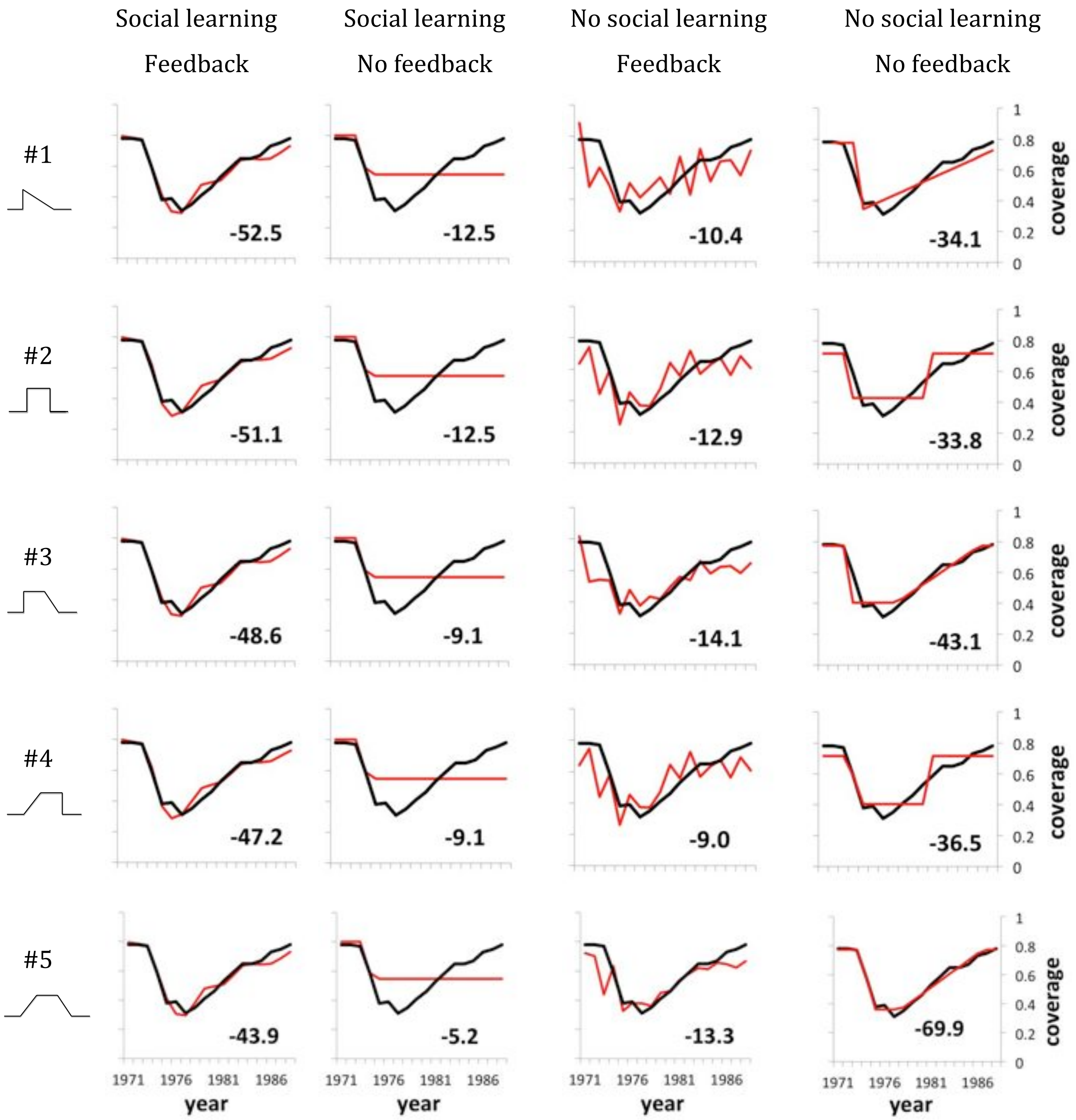

Social learning
Feedback
Social learning
No feedback
No social learning
Feedback
No social learning
No feedback
#1
-52.5
-12.5
-10.4
-34.1
#2
-51.1
-12.5
-12.9
-33.8
#3
-48.6
-9.1
-14.1
-43.1
#4
-47.2
-9.1
-9.0
-36.5
#5
-43.9
-5.2
-13.3
-69.9
coverage
0
0.2
0.4
0.6
0.8
1
1971
1976
1981
1986
year
