# Supplementary material for: Evolutionary Game Theory and Social Learning Can Determine How Vaccine Scares Unfold
Source: PLoS Comput Biol. 2012 Apr 5;8(4):e1002452. doi: 10.1371/journal.pcbi.1002452 (PMC3320575; doi:10.1371/journal.pcbi.1002452)
Supplement: Figure S17 — Best fit of behaviour-incidence model (red) to 10 sets of correlated white noise data and (black), for risk evolution curve #6 Also shown are log of maximum likelihood function and AICc of best fit (figure inset). Vertical scales range from 0.7 to 1.0; horizontal 1995 to 2009. Model was not fitted to vaccine coverage data using this risk evolution curve since a constant perceived vaccine penalty (curve #6) would correspond to no vaccine scare having occurred. (PDF) [file pcbi.1002452.s017.pdf]

Best fit of model to MMR vaccine coverage data	
N/A	
**Best fit of model to correlated white noise data**	
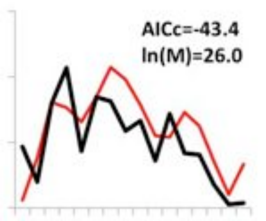	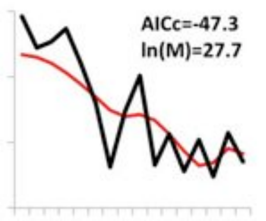
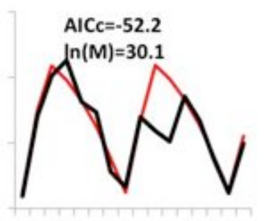	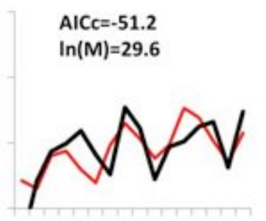
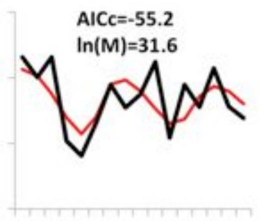	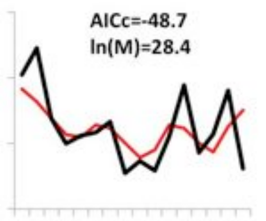
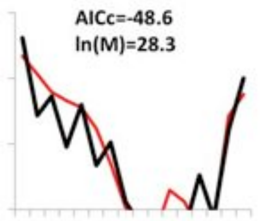	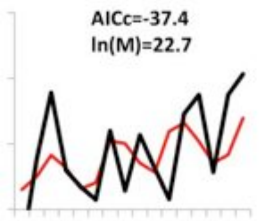
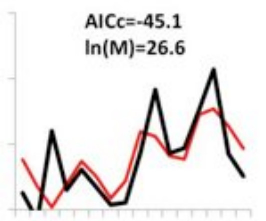	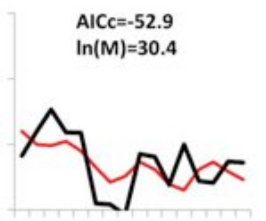
