# Supplementary material for: Evolutionary Game Theory and Social Learning Can Determine How Vaccine Scares Unfold
Source: PLoS Comput Biol. 2012 Apr 5;8(4):e1002452. doi: 10.1371/journal.pcbi.1002452 (PMC3320575; doi:10.1371/journal.pcbi.1002452)
Supplement: Figure S18 — PSA Results, Pertussis, t fit from 1975 to 1988. Solid black line represents vaccine coverage/incidence data for t≤t fit; dashed black line represents vaccine coverage/incidence data for t>t fit (data from years t≤t fit were used to fit model and produce model extrapolation to t>t fit); dotted blue line represents the best fit of model to data for given value of t fit; thin red lines represent 50 Monte Carlo samples for a given value of t fit. (PDF) [file pcbi.1002452.s018.pdf]

Supporting Figure 18: PSA Results, Pertussis, t_{fit} from 1975 to 1988*.

$t_{\mathrm{fit}} = 1975$

$t_{\mathrm{fit}} = 1976$

$t_{\mathrm{fit}} = 1977$

$t_{\mathrm{fit}} = 1978$

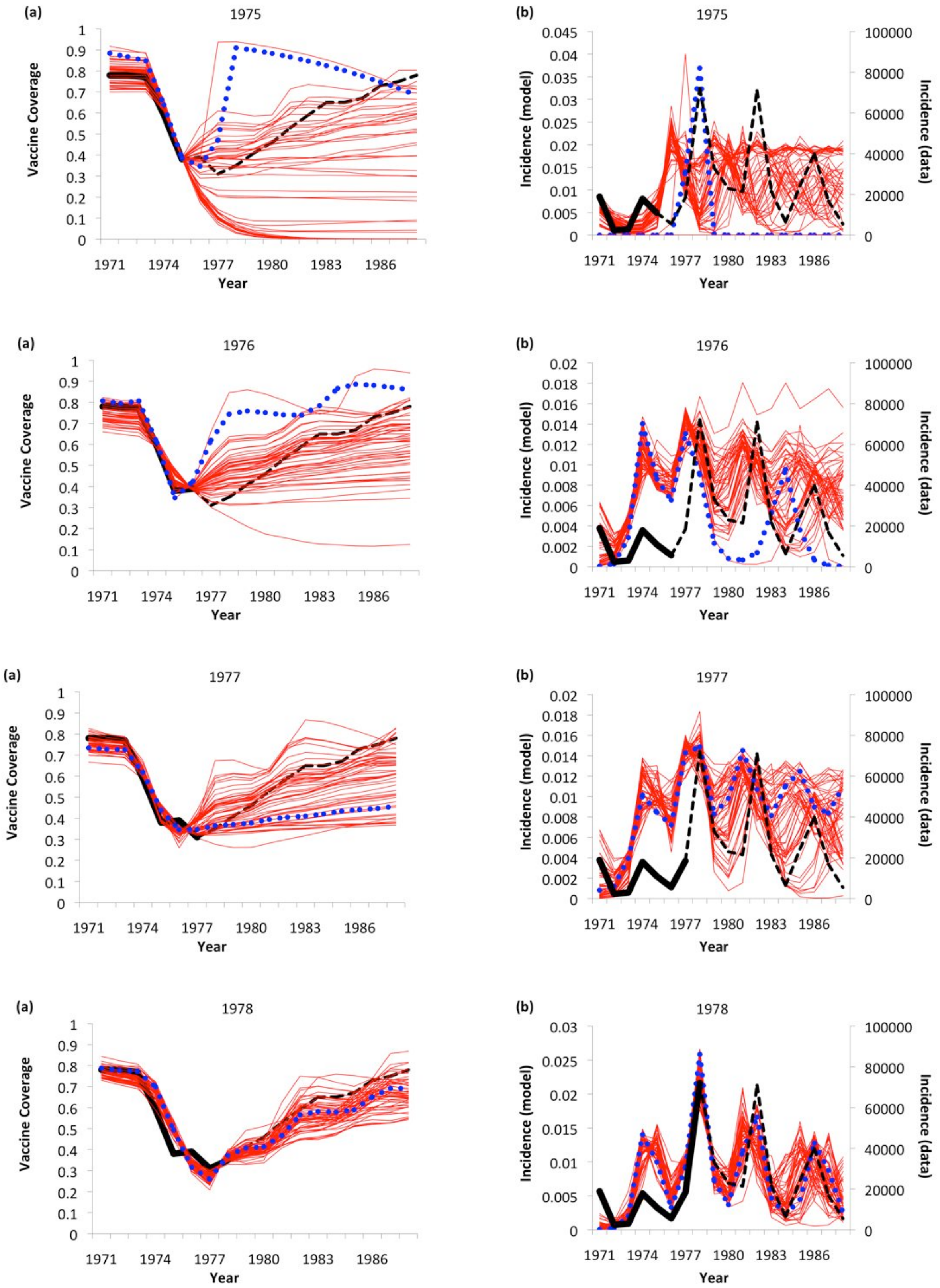

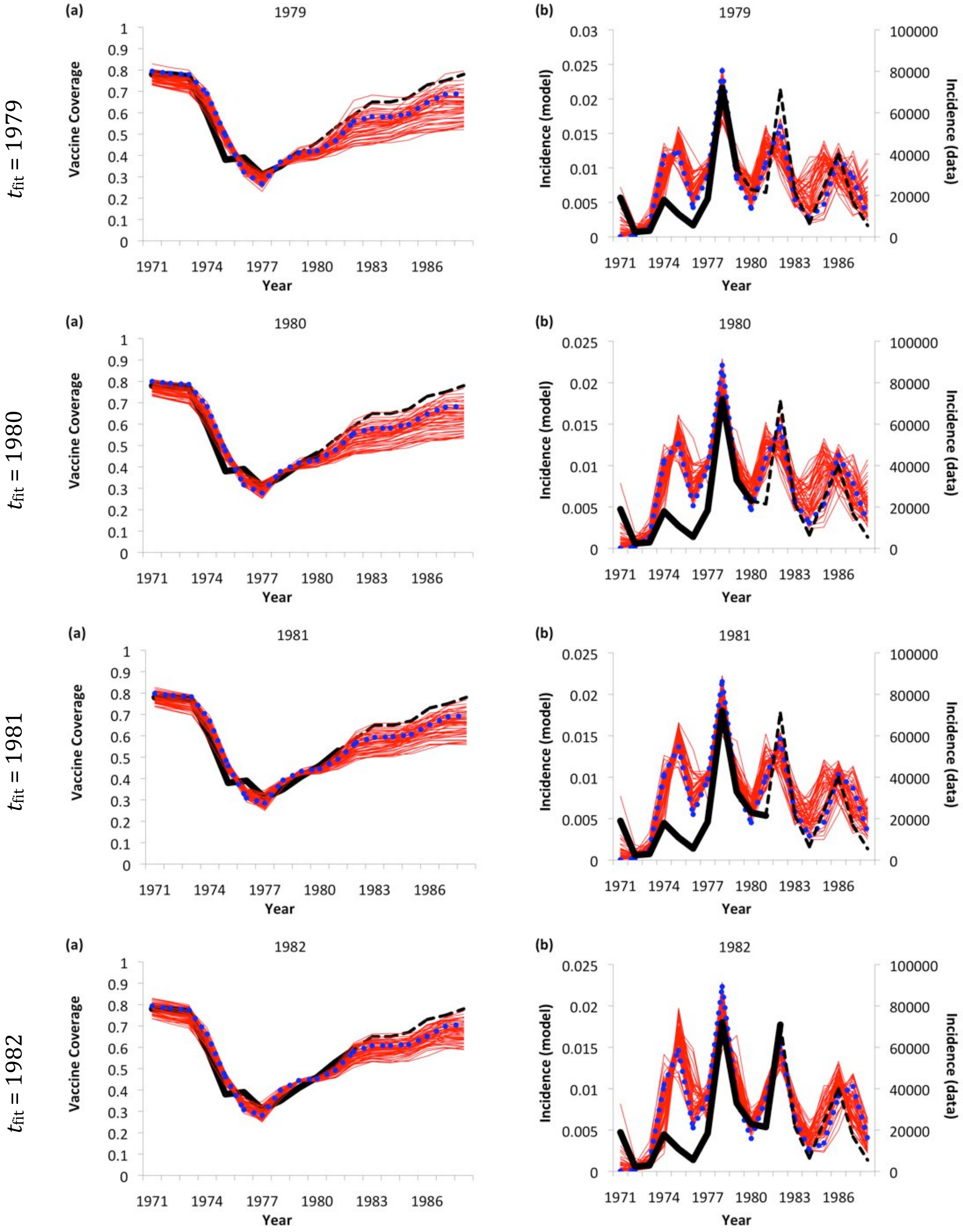
$t_{\mathrm{fit}} = 1979$
$t_{\mathrm{fit}} = 1980$
$t_{\mathrm{fit}} = 1981$
$t_{\mathrm{fit}} = 1982$
(a)
(b)
1979
1980
1981
1982
Vaccine Coverage
Incidence (model)
Incidence (data)
Year
1971
1974
1977
1980
1983
1986

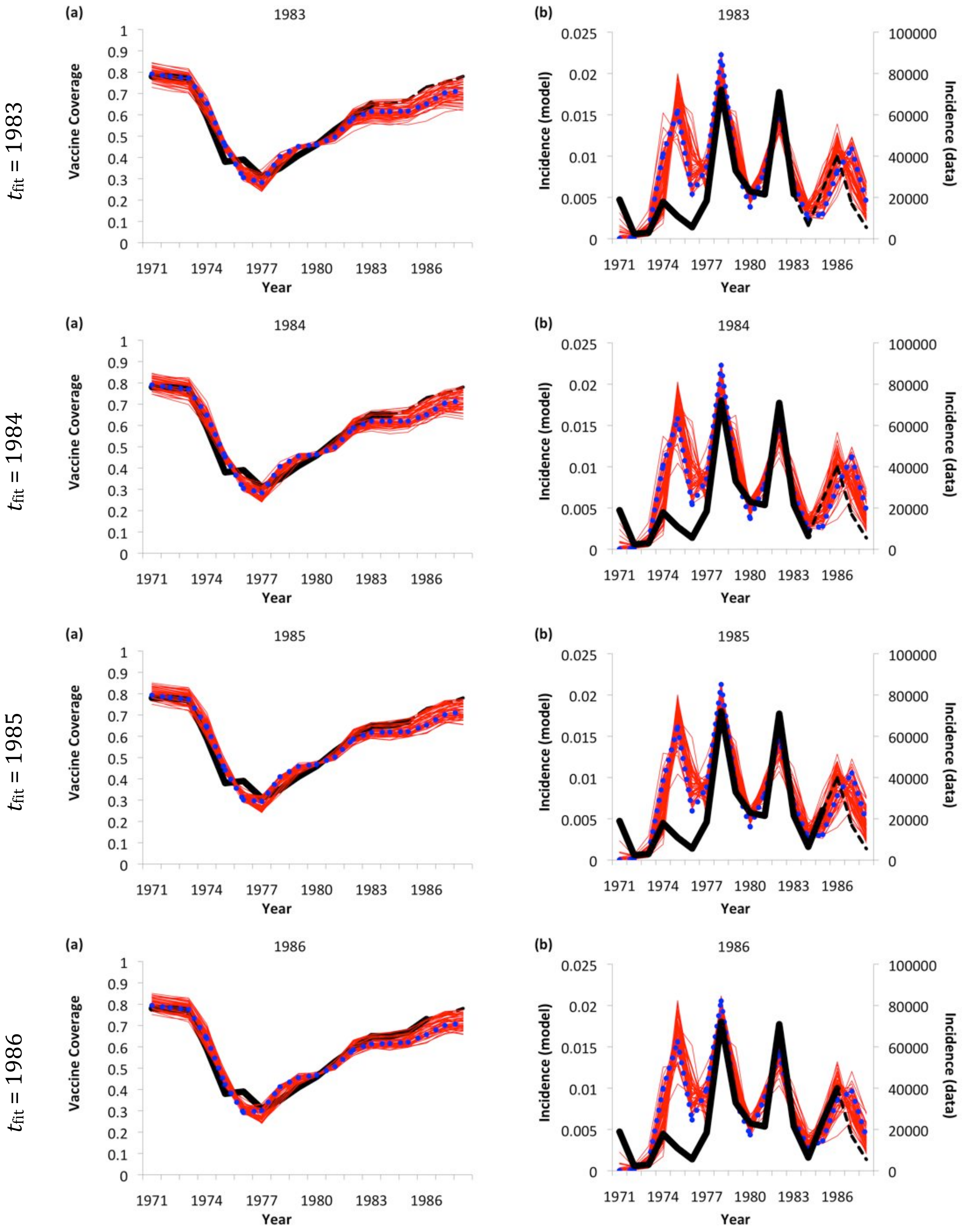
$t_{\mathrm{fit}} = 1983$
(a)
1983
Vaccine Coverage
Year
(b)
1983
Incidence (model)
Incidence (data)
Year
$t_{\mathrm{fit}} = 1984$
(a)
1984
Vaccine Coverage
Year
(b)
1984
Incidence (model)
Incidence (data)
Year
$t_{\mathrm{fit}} = 1985$
(a)
1985
Vaccine Coverage
Year
(b)
1985
Incidence (model)
Incidence (data)
Year
$t_{\mathrm{fit}} = 1986$
(a)
1986
Vaccine Coverage
Year
(b)
1986
Incidence (model)
Incidence (data)
Year

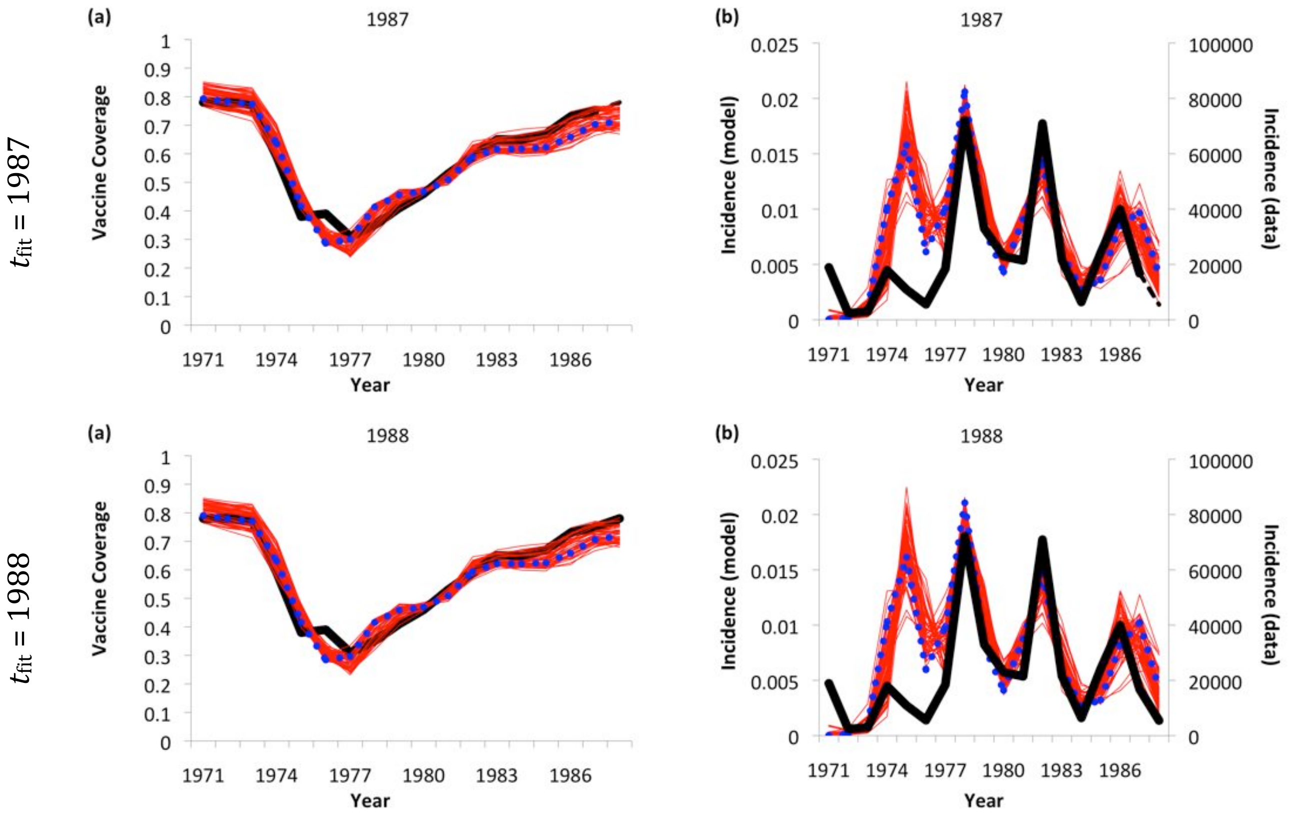

* Supporting Figure 18. Solid black line represents vaccine coverage/incidence data for $t \leq t_{\mathrm{fit}}$; dashed black line represents vaccine coverage/incidence data for $t > t_{\mathrm{fit}}$ (data from years $t \leq t_{\mathrm{fit}}$ were used to fit model and produce model extrapolation to $t > t_{\mathrm{fit}}$); dotted blue line represents the best fit of model to data for given value of t_{fit}; thin red lines represent 50 Monte Carlo samples for a given value of t_{fit}.
