# Supplementary material for: Evolutionary Game Theory and Social Learning Can Determine How Vaccine Scares Unfold
Source: PLoS Comput Biol. 2012 Apr 5;8(4):e1002452. doi: 10.1371/journal.pcbi.1002452 (PMC3320575; doi:10.1371/journal.pcbi.1002452)
Supplement: Figure S20 — PSA for MMR, t fit values from 1997 to 2009. Solid black line represents vaccine coverage/incidence data for t≤t fit; dashed black line represents vaccine coverage/incidence data for t>t fit (data from years t≤t fit were used to fit model and produce model extrapolation to t>t fit); dotted blue line represents the best fit of model to data for given value of t fit; thin red lines represent 50 Monte Carlo samples for a given value of t fit. (PDF) [file pcbi.1002452.s020.pdf]

<u>Supporting Figure 20</u>: PSA for MMR, t_{fit} values from 1997 to 2009*

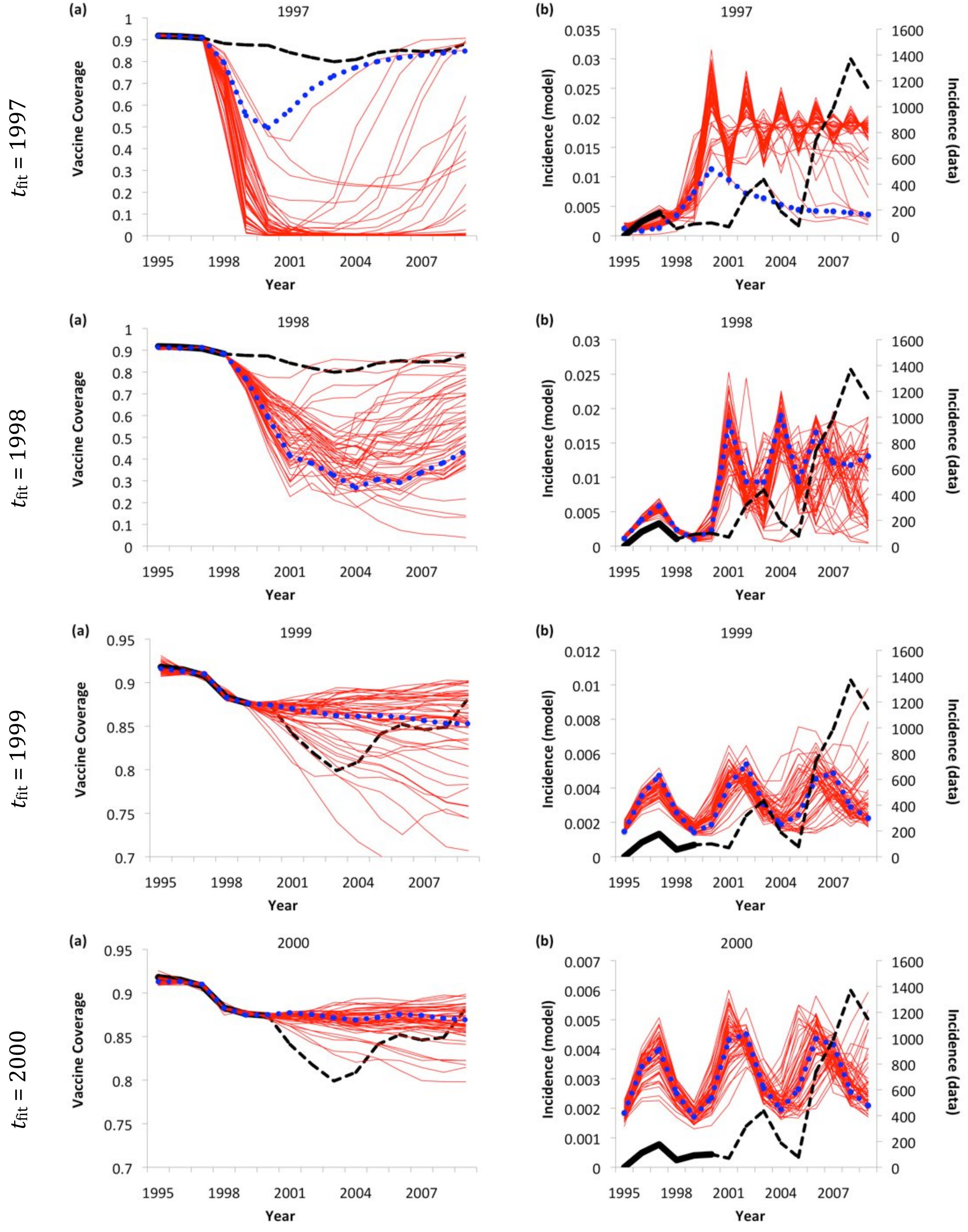

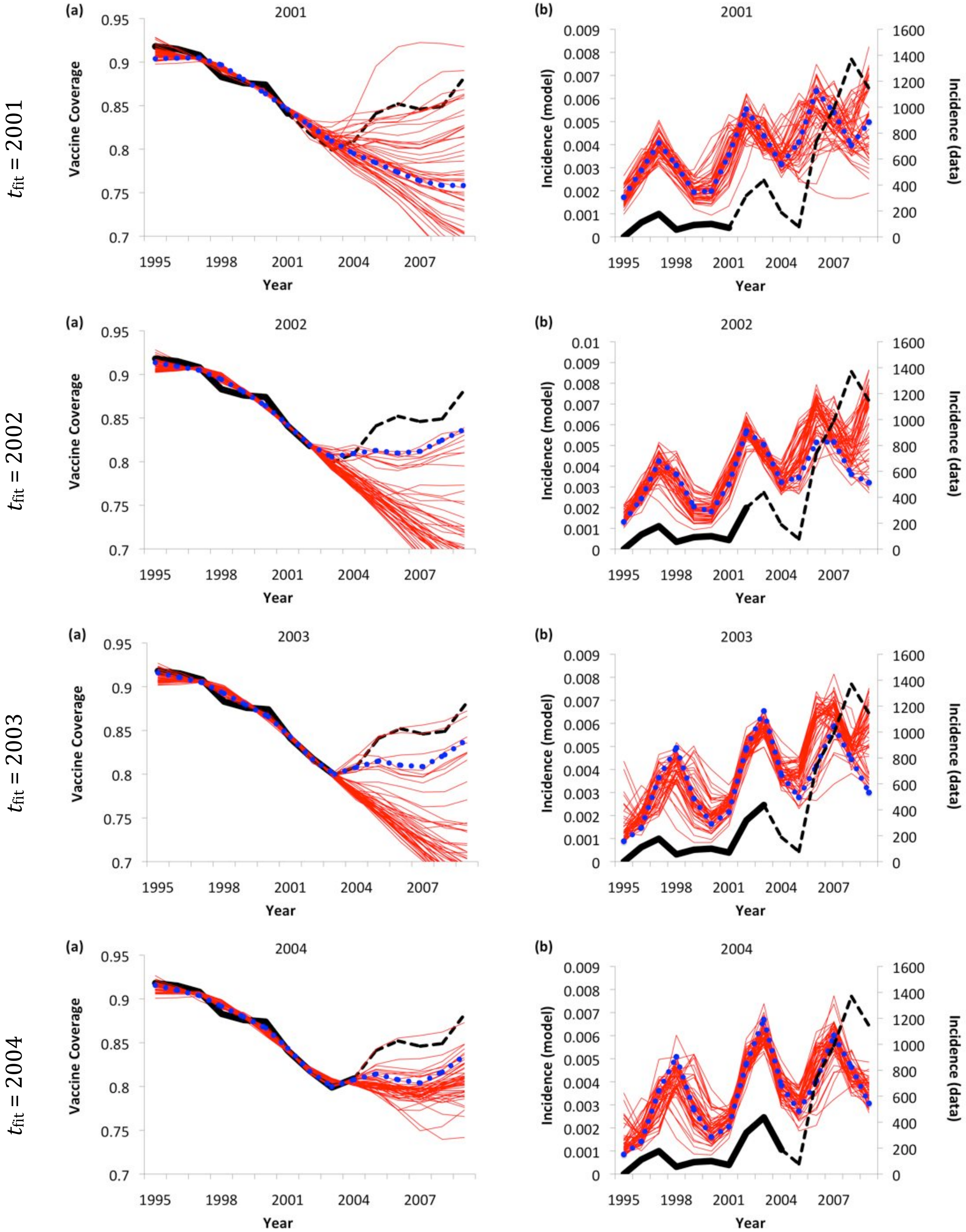
t_{fit} = 2001
t_{fit} = 2002
t_{fit} = 2003
t_{fit} = 2004
(a)
(b)
2001
2002
2003
2004
Vaccine Coverage
Incidence (model)
Incidence (data)
Year
1995
1998
2001
2004
2007

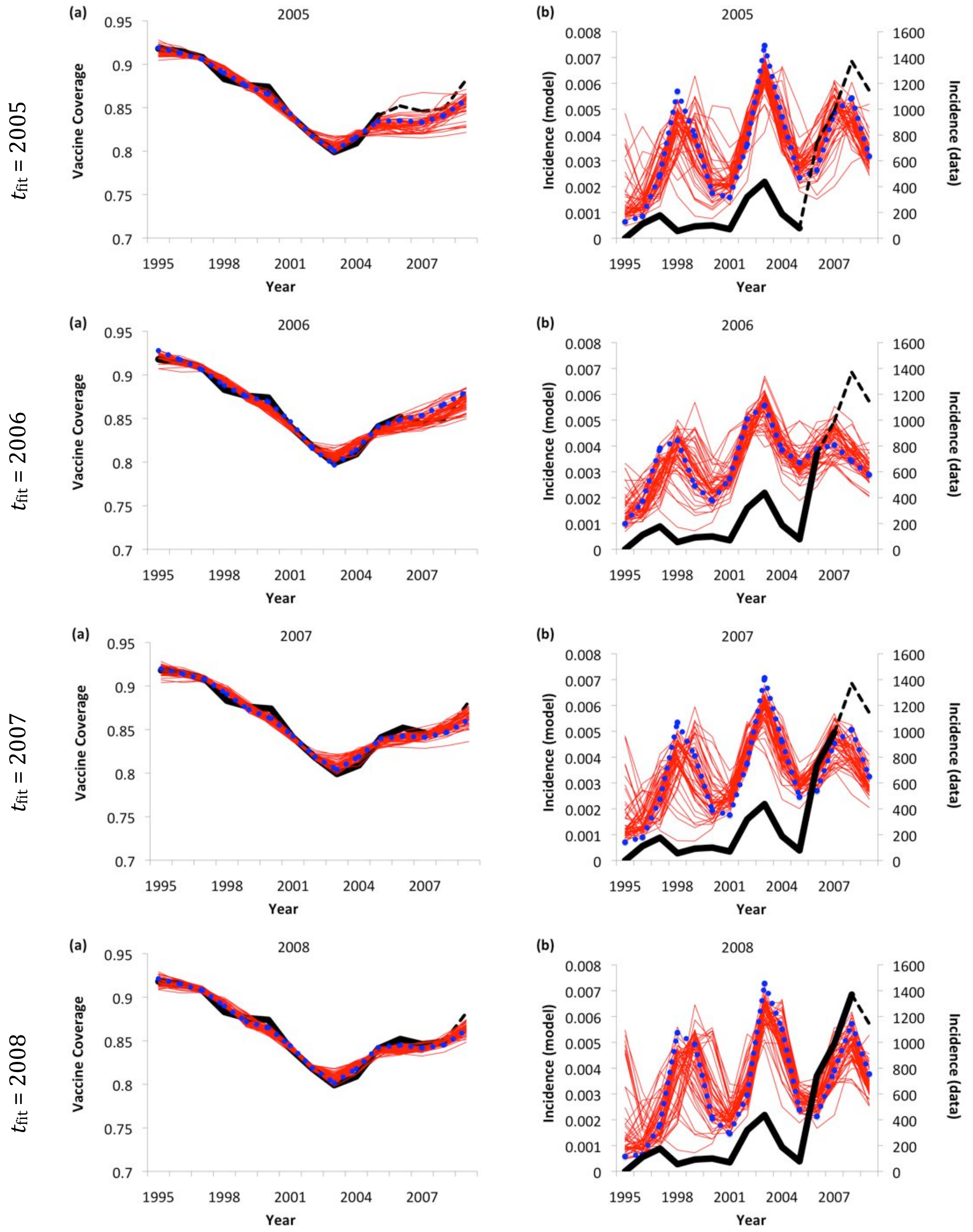
t_{fit} = 2005
(a)
2005
Vaccine Coverage
Year
(b)
2005
Incidence (model)
Incidence (data)
Year
t_{fit} = 2006
(a)
2006
Vaccine Coverage
Year
(b)
2006
Incidence (model)
Incidence (data)
Year
t_{fit} = 2007
(a)
2007
Vaccine Coverage
Year
(b)
2007
Incidence (model)
Incidence (data)
Year
t_{fit} = 2008
(a)
2008
Vaccine Coverage
Year
(b)
2008
Incidence (model)
Incidence (data)
Year

$t_{\mathrm{fit}} = 2009$

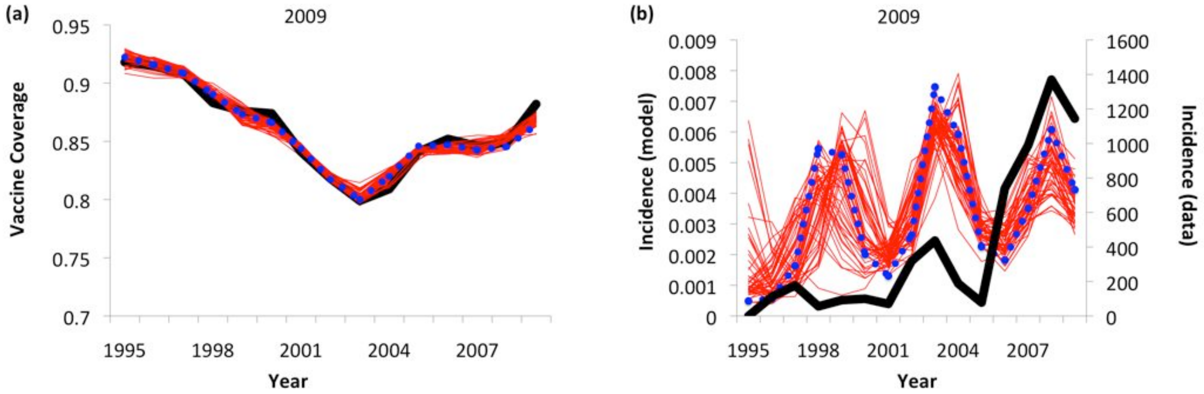

* Supporting Figure 20. Solid black line represents vaccine coverage/incidence data for $t \leq t_{\mathrm{fit}}$; dashed black line represents vaccine coverage/incidence data for $t > t_{\mathrm{fit}}$ (data from years $t \leq t_{\mathrm{fit}}$ were used to fit model and produce model extrapolation to $t > t_{\mathrm{fit}}$); dotted blue line represents the best fit of model to data for given value of t_{fit}; thin red lines represent 50 Monte Carlo samples for a given value of t_{fit}.
