# Supplementary material for: Evolutionary Game Theory and Social Learning Can Determine How Vaccine Scares Unfold
Source: PLoS Comput Biol. 2012 Apr 5;8(4):e1002452. doi: 10.1371/journal.pcbi.1002452 (PMC3320575; doi:10.1371/journal.pcbi.1002452)
Supplement: Figure S21 — Bootstrapping Results for MMR, t fit from 1997 to 2009. Solid black line represents vaccine coverage/incidence data for t≤t fit; dashed black line represents vaccine coverage/incidence data for t>t fit (data from years t≤t fit were used to fit model and produce model extrapolation to t>t fit); dotted blue line represents the best fit of model to data for given value of t fit; thin red lines represent 50 bootstrap samples for a given value of t fit. (PDF) [file pcbi.1002452.s021.pdf]

Supporting Figure 21. Bootstrapping Results for MMR, t_{fit} from 1997 to 2009*

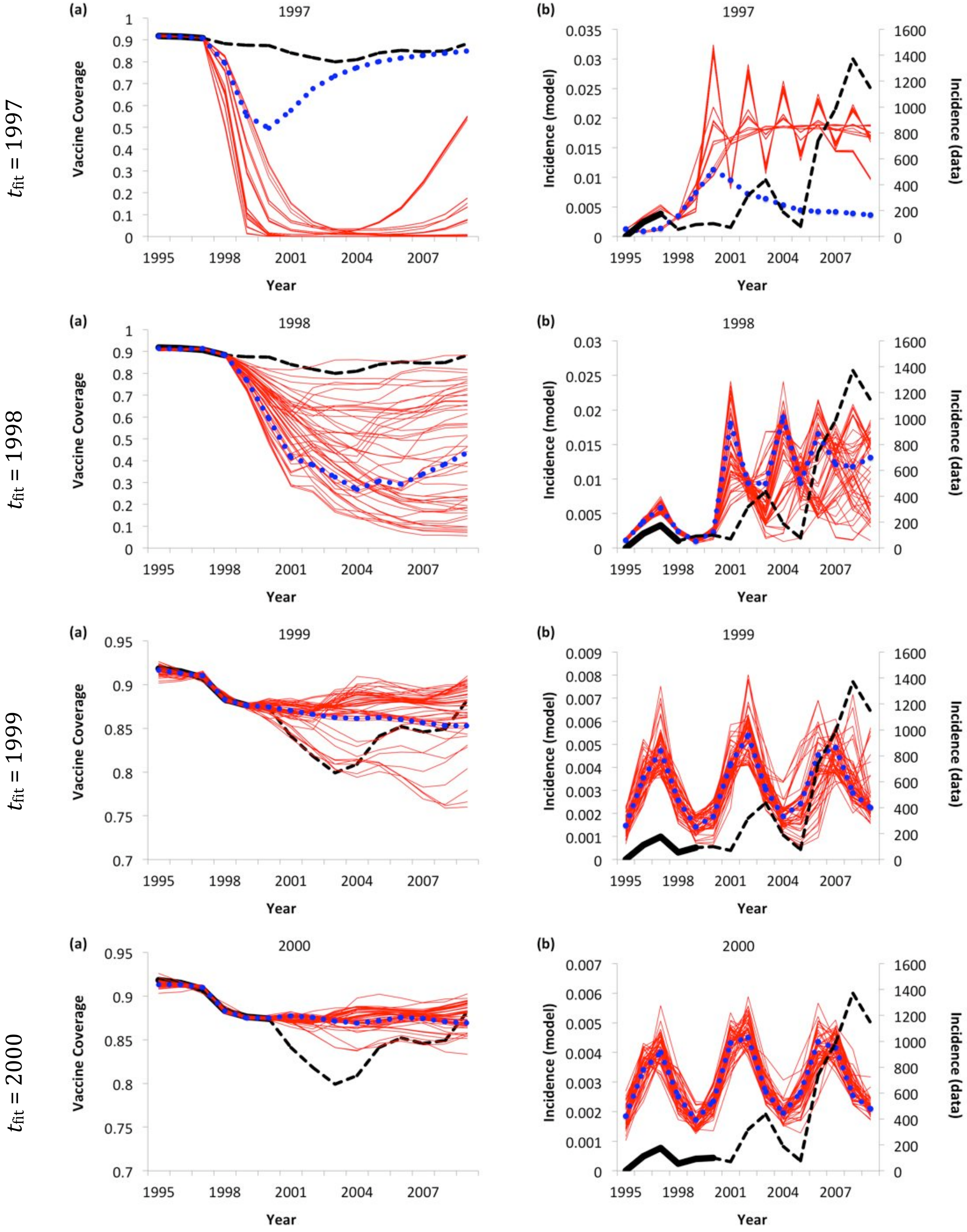

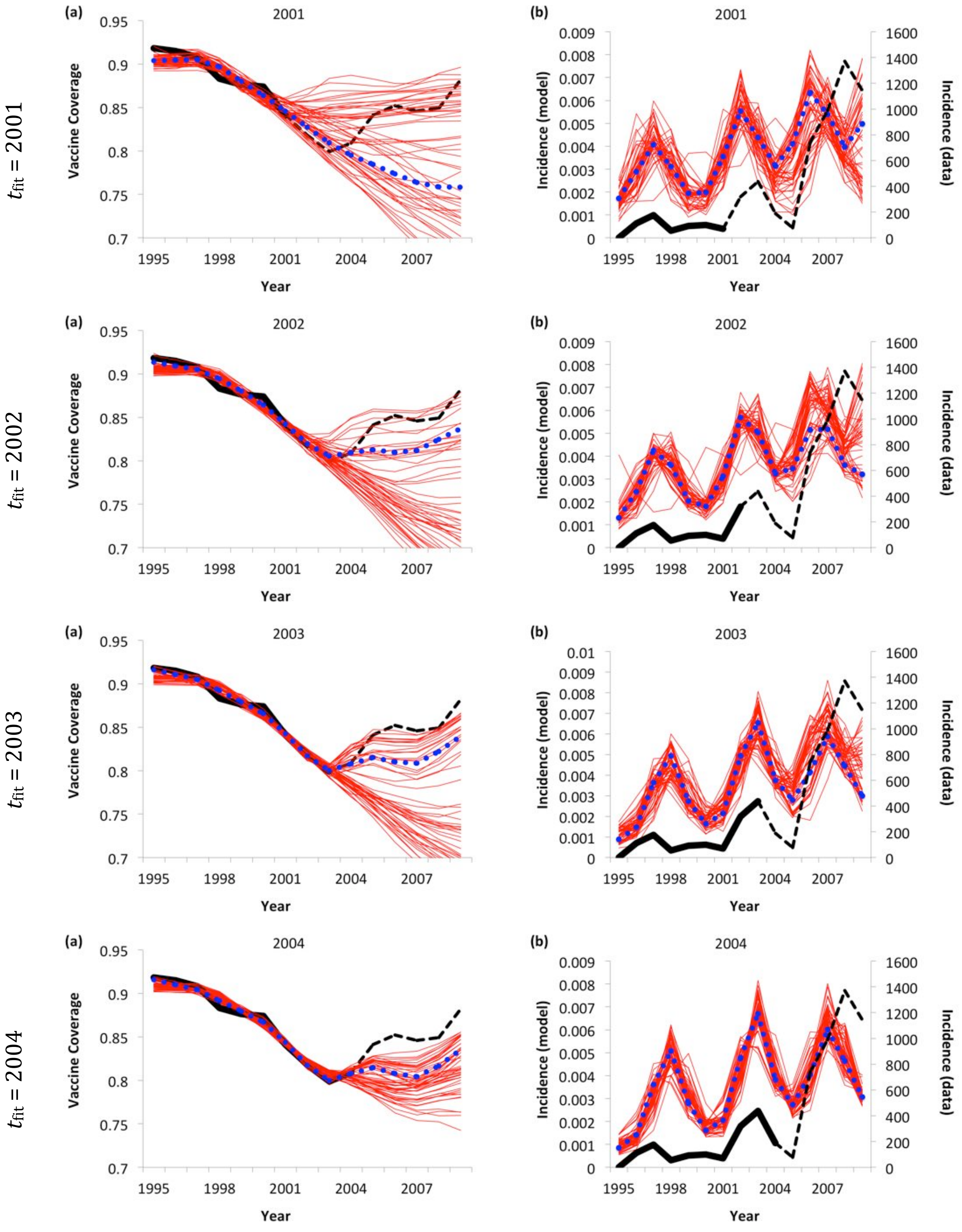
$t_{\text{fit}} = 2001$
$t_{\text{fit}} = 2002$
$t_{\text{fit}} = 2003$
$t_{\text{fit}} = 2004$
(a)
(b)
2001
2002
2003
2004
Vaccine Coverage
Incidence (model)
Incidence (data)
Year
0.95
0.9
0.85
0.8
0.75
0.7
0.01
0.009
0.008
0.007
0.006
0.005
0.004
0.003
0.002
0.001
0
1600
1400
1200
1000
800
600
400
200
1995
1998
2001
2004
2007

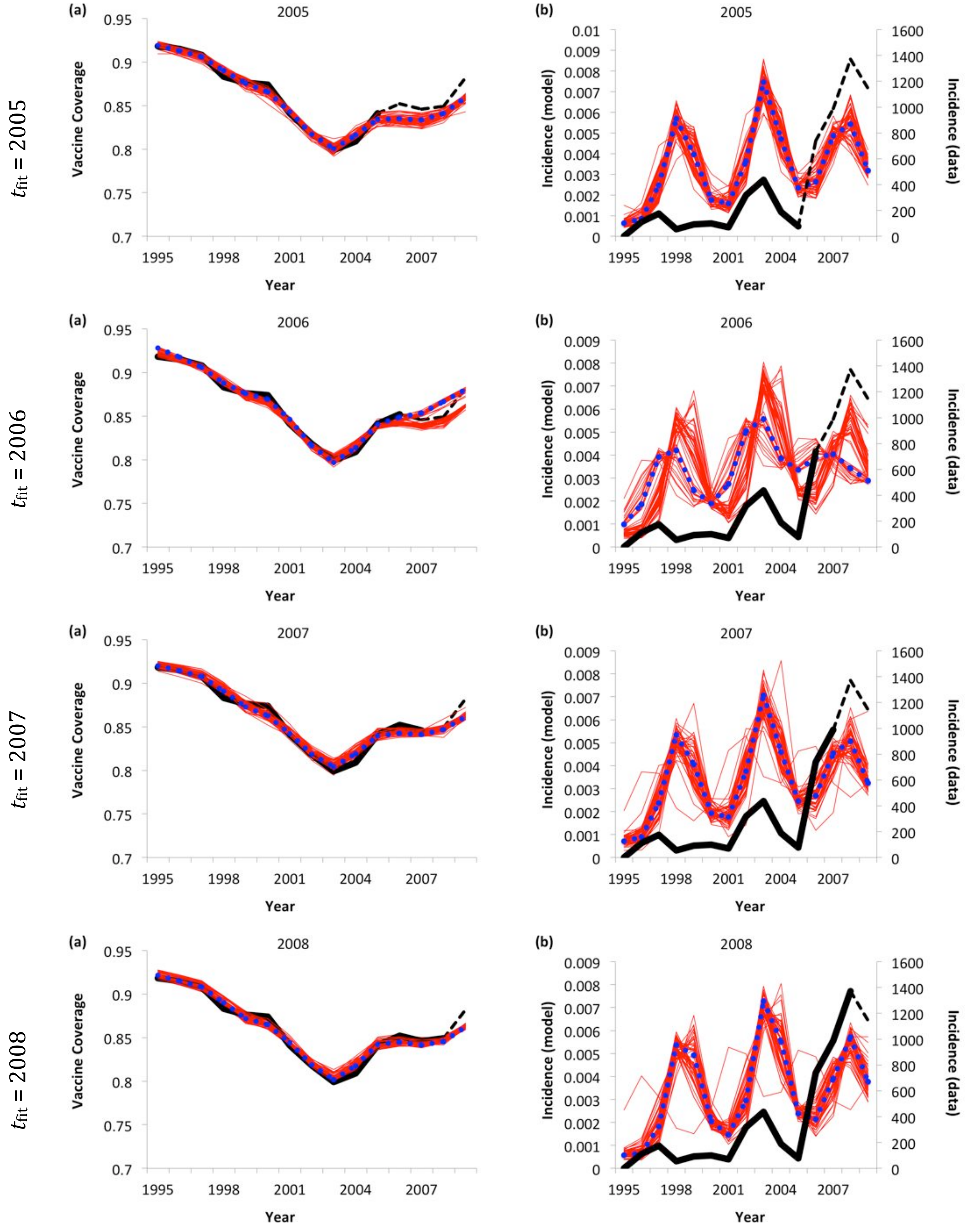
$t_{\text{fit}} = 2005$
$t_{\text{fit}} = 2006$
$t_{\text{fit}} = 2007$
$t_{\text{fit}} = 2008$
(a)
2005
2006
2007
2008
Vaccine Coverage
Year
1995
1998
2001
2004
2007
0.7
0.75
0.8
0.85
0.9
0.95
(b)
Incidence (model)
Incidence (data)
0
0.001
0.002
0.003
0.004
0.005
0.006
0.007
0.008
0.009
0.01
200
400
600
800
1000
1200
1400
1600

$t_{\text{fit}} = 2009$

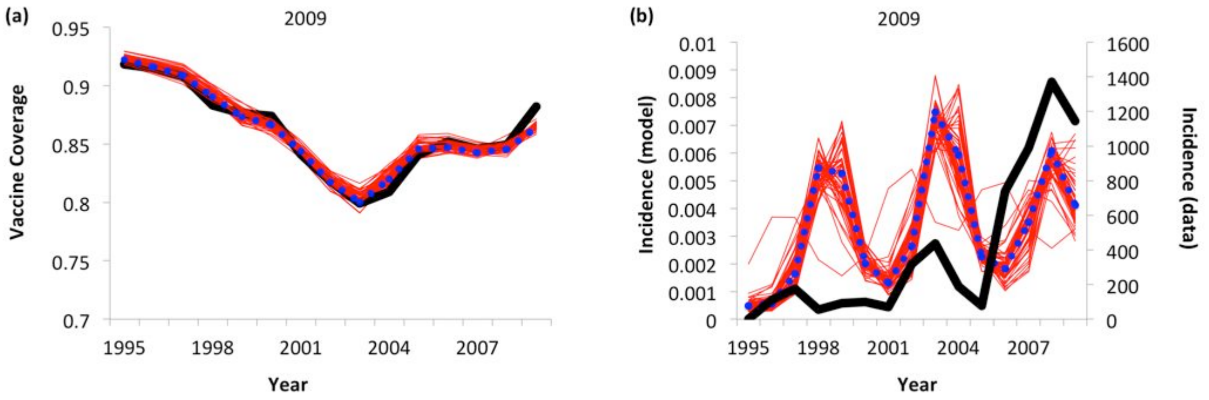

* Supporting Figure 21. Solid black line represents vaccine coverage/incidence data for $t \leq t_{\text{fit}}$; dashed black line represents vaccine coverage/incidence data for $t > t_{\text{fit}}$ (data from years $t \leq t_{\text{fit}}$ were used to fit model and produce model extrapolation to $t > t_{\text{fit}}$); dotted blue line represents the best fit of model to data for given value of t_{fit}; thin red lines represent 50 bootstrap samples for a given value of t_{fit}.
