# Supplementary material for: Evolutionary Game Theory and Social Learning Can Determine How Vaccine Scares Unfold
Source: PLoS Comput Biol. 2012 Apr 5;8(4):e1002452. doi: 10.1371/journal.pcbi.1002452 (PMC3320575; doi:10.1371/journal.pcbi.1002452)
Supplement: Table S1 — Confidence interval of fitted parameters for all 5 risk evolution curves models for the behavioral model with social learning and feedback, derived from bootstrapping. (PDF) [file pcbi.1002452.s022.pdf]

Model - 1	κ		ω_{pre}		σ		$D_{decrease}$	
	CI L	CI U	CI L	CI U	CI L	CI U	CI L	CI U
Pertussis	6.6024E-06	8.152E-06	1900.005	2000.03	50.196	51.361	6.567	7.818
Measles	6.00E-05	1.4478E-04	13.655	30	115	126.9031	7	9

Model - 2	κ		ω_{pre}		σ		D_{max}	
	CI L	CI U	CI L	CI U	CI L	CI U	CI L	CI U
Pertussis	6.4246E-06	9.2381E-06	996.2821	997.0840	59.1563	70.0086	5.031	5.600
Measles	1.00E-04	1.4173E-04	13.4445	20	100.1575	100.9820	5.0341	7.1887

Model - 3	κ		ω_{pre}		σ		D_{max}		$D_{decrease}$	
	CI L	CI U	CI L	CI U	CI L	CI U	CI L	CI U	CI L	CI U
Pertussis	5.2115E-06	7.3429E-06	1900.032	2000.091	49.706	50.3385	1.6471	3.2016	0.9924	4.3161
Measles	1.00E-04	2.2804E-04	10	20	90	101.001<br>6	3.8123	5.5	1	3

Model - 4	κ		ω_{pre}		σ		$D_{increase}$		D_{max}	
	CI L	CI U	CI L	CI U	CI L	CI U	CI L	CI U	CI L	CI U
Pertussis	5.3689E-06	8.5201E-06	1999.7	2560.71	27.5905	40.5974	0.5	1.9883	3.0117	4.5059
Measles	1.4929E-04	2.9197E-04	9	12.2656	90	100.223<br>2	2.8750	4	3.875	4.125

Model - 5	κ		ω_{pre}		σ		$D_{increase}$		D_{max}		$D_{decrease}$	
	CI L	CI U	CI L	CI U	CI L	CI U	CI L	CI U	CI L	CI U	CI L	CI U
Pertussis	5.2865E-06	7.7883E-06	1999.8	2000.1	44.0842	50.8694	1.9708	2.2401	1	1.3059	1	2.0387
Measles	1.00E-04	1.6856E-04	11.0685	22.0012	98.2717	100.394<br>3	2.0253	4.1387	1.2606	3	`1	3
