# Supplementary material for: Evolutionary Game Theory and Social Learning Can Determine How Vaccine Scares Unfold
Source: PLoS Comput Biol. 2012 Apr 5;8(4):e1002452. doi: 10.1371/journal.pcbi.1002452 (PMC3320575; doi:10.1371/journal.pcbi.1002452)
Supplement: Table S2 — Fitting results for behavioral model with social learning and feedback under 5 risk evolution curves. (PDF) [file pcbi.1002452.s023.pdf]

<u>Supporting Table 2</u>: Fitting results for behavioral model with social learning and feedback under 5 risk evolution curves.

	Model-1	
	**Pertussis**	**Measles**
**κ**	7.00E-06	9.18E-05
$\omega_{\mathbf{pre}}$	2000	20
σ	50.3262	125.3202
$D_{\mathbf{decrease}}$	6.9434	8
**MLE**	5.82E+33	8.80E+40
**GOF**	0.9529	0.8673
**AICc**	- 144.4167	-176.5557

	Model-2	
	**Pertussis**	**Measles**
**K**	7.84E-06	1.15E-04
$\omega_{\mathbf{pre}}$	996.6676	13.9864
σ	66.9777	100.3957
$D_{\mathbf{max}}$	5.2	6.5
**MLE**	1.77E+30	2.09E+42
**GOF**	0.9261	0.8926
**AICc**	-128.2147	-182.8945

	Model-3	
	**Pertussis**	**Measles**
**κ**	6.15E-06	1.05E-04
$\omega_{\mathbf{pre}}$	2000	16
σ	49.5471	100.608
$D_{\mathbf{max}}$	2.6391	4.9597
$D_{\mathbf{decrease}}$	2.1302	2.0389
**MLE**	9.61E+33	8.87E+42
**GOF**	0.9542	0.9024
**AICc**	-141.4952	-181.115

	Model-4	
	**Pertussis**	**Measles**
**K**	7.96E-06	1.94E-04
$\omega_{\mathbf{pre}}$	2364.4	11
σ	27.6089	100.0988
$D_{\mathbf{increase}}$	1	3
$D_{\mathbf{max}}$	4.0235	4
**MLE**	3.11E+30	1.29E+37
**GOF**	0.9284	0.761
**AICc**	-125.4280	-154.238

	Model-5	
	**Pertussis**	**Measles**
**κ**	6.28E-06	1.11E-04
$\omega_{\mathbf{pre}}$	2000	19.7
σ	48.8694	99.9456
$D_{\mathbf{increase}}$	2	3.4374
$D_{\mathbf{max}}$	1.1528	1.3556
$D_{\mathbf{decrease}}$	1.1094	1.6089
**MLE**	3.47E+34	7.98E+40
**GOF**	0.9573	0.8665
**AICc**	-139.4262	-165.8595
