# Supplementary material for: Evolutionary Game Theory and Social Learning Can Determine How Vaccine Scares Unfold
Source: PLoS Comput Biol. 2012 Apr 5;8(4):e1002452. doi: 10.1371/journal.pcbi.1002452 (PMC3320575; doi:10.1371/journal.pcbi.1002452)
Supplement: Table S3 — Fitting results for behavioral model with social learning but no feedback under 5 risk evolution curves. (PDF) [file pcbi.1002452.s024.pdf]

	Model-1	
	**Pertussis**	**Measles**
ω_{pre}	8.81E-03	1.02E-03
σ	50.0277	200.0474
$D_{decrease}$	3.3408	6.7691
**MLE**	2.25E+11	5.59E+35
**GOF**	0.1715	0.7054
**AICc**	-41.2014	-152.6243

	Model-2	
	**Pertussis**	**Measles**
ω_{pre}	1.86E-03	5.95E-04
σ	114.8997	199.9999
D_{max}	4.75	6.1842
**MLE**	5.05E+10	2.34E+34
**GOF**	0.0998	0.6359
**AICc**	-38.2125	-146.2721

	Model-3	
	**Pertussis**	**Measles**
ω_{pre}	7.70E-03	7.52E-04
σ	49.279	200.0022
D_{max}	1.3517	4.3253
$D_{decrease}$	1.6484	1.0205
**MLE**	3.80E+11	1.02E+36
**GOF**	0.1953	0.717
**AICc**	-38.3254	-149.1646

	Model-4	
	**Pertussis**	**Measles**
ω_{pre}	1.59E-03	5.95E-04
σ	100.2588	200.0811
$D_{increase}$	1.2344	1
D_{max}	3	5
**MLE**	6.53E+10	2.34E+34
**GOF**	0.1126	0.6359
**AICc**	-34.8031	-141.606

	Model-5	
	**Pertussis**	**Measles**
ω_{pre}	4.44E-03	8.79E-04
σ	49.7754	199.9777
$D_{increase}$	1.0433	2.3671
D_{max}	1.9822	1
$D_{decrease}$	1	2.4731
**MLE**	2.90E+11	4.46E+35
**GOF**	0.1831	0.7009
**AICc**	-33.1489	-141.6702
