# Supplementary material for: Evolutionary Game Theory and Social Learning Can Determine How Vaccine Scares Unfold
Source: PLoS Comput Biol. 2012 Apr 5;8(4):e1002452. doi: 10.1371/journal.pcbi.1002452 (PMC3320575; doi:10.1371/journal.pcbi.1002452)
Supplement: Table S4 — Fitting results for behavioral model with feedback but no social learning under 5 risk evolution curves. (PDF) [file pcbi.1002452.s025.pdf]

<u>Supporting Table 4</u>: Fitting results for behavioral model with social learning but no feedback under 5 risk evolution curves.

	**Model-1**	
	**Pertussis**	**Measles**
ω_{pre}	0.1	0.1
**Gamma**	1.59E-05	0.0011
σ	0.001	0.001
$D_{decrease}$	5	10
**MLE**	2.58E-08	4.01E-11
**GOF**	-8.3434	-350.196
**AICc**	46.0212	59.8798

	**Model-2**	
	**Pertussis**	**Measles**
ω_{pre}	0.1	0.1
**Gamma**	1.58E-05	0.0011
σ	0.001	0.001
D_{max}	4	4
**MLE**	3.59E-08	3.95E-11
**GOF**	-8.1746	-350.5707
**AICc**	42.0021	56.0936

	**Model-3**	
	**Pertussis**	**Measles**
ω_{pre}	0.1	0.1
**Gamma**	1.53E-05	0.0011
σ	0.001	0.001
D_{max}	2.9806	9.552
$D_{decrease}$	6.0194	0.3218
**MLE**	2.63E-08	1.22E-10
**GOF**	-8.3348	-325.114
**AICc**	49.911	62.3235

	**Model-4**	
	**Pertussis**	**Measles**
ω_{pre}	0.1	0.2477
**Gamma**	1.53E-05	0.0011
σ	0.001	0.001
$D_{increase}$	3	2.4293
D_{max}	6.002	8.5707
**MLE**	3.88E-08	3.00E-10
**GOF**	-8.1339	-306.07
**AICc**	49.1276	60.5184

	**Model-5**	
	**Pertussis**	**Measles**
ω_{pre}	0.1	0.1
**Gamma**	1.38E-05	0.0011
σ	0.001	0.001
$D_{increase}$	3	3.0011
D_{max}	10	6.3901
$D_{decrease}$	10	0.0927
**MLE**	1.25E-07	9.47E-11
**GOF**	-7.5597	-330.6411
