# Supplementary material for: Evolutionary Game Theory and Social Learning Can Determine How Vaccine Scares Unfold
Source: PLoS Comput Biol. 2012 Apr 5;8(4):e1002452. doi: 10.1371/journal.pcbi.1002452 (PMC3320575; doi:10.1371/journal.pcbi.1002452)
Supplement: Table S5 — Fitting results for behavioral model with no feedback and no social learning under 5 risk evolution curves. (PDF) [file pcbi.1002452.s026.pdf]

| | Model-1 | |
|---|---|---|
| | Pertussis | Measles |
| $\omega_{pre}$ | 3.39E-01 | 0.1131 |
| $\sigma$ | 1.7121 | 1.633 |
| $D_{decrease}$ | 10 | 10 |
| MLE | 2.07E+12 | 1.25E+32 |
| GOF | 0.2677 | 0.4839 |
| AICc | -49.0039 | -139.6246 |

| | Model-2 | |
|---|---|---|
| | Pertussis | Measles |
| $\omega_{pre}$ | 2.76E-01 | 0.1197 |
| $\sigma$ | 1.8896 | 1.4918 |
| $D_{max}$ | 9.5 | 4.5 |
| MLE | 2.80E+16 | 4.75E+33 |
| GOF | 0.5682 | 0.5951 |
| AICc | -68.0254 | -146.9046 |

| | Model-3 | |
|---|---|---|
| | Pertussis | Measles |
| $\omega_{pre}$ | 2.20E-01 | 0.1 |
| $\sigma$ | 2.4675 | 1.6674 |
| $D_{max}$ | 6.09917 | 6.6058 |
| $D_{decrease}$ | 8.2936 | 3.6602 |
| MLE | 1.27E+18 | 5.06E+35 |
| GOF | 0.6507 | 0.7034 |
| AICc | -72.2956 | -152.4245 |

| | Model-4 | |
|---|---|---|
| | Pertussis | Measles |
| $\omega_{pre}$ | 0.3197 | 0.0999 |
| $\sigma$ | 1.9864 | 1.7289 |
| $D_{increase}$ | 2.2817 | 6.6458 |
| $D_{max}$ | 4.1091 | 4.3542 |
| MLE | 4.59E+19 | 2.28E+36 |
| GOF | 0.7138 | 0.7318 |
| AICc | -79.4683 | -155.438 |

| | Model-5 | |
|---|---|---|
| | Pertussis | Measles |
| $\omega_{pre}$ | 0.2269 | 0.0871 |
| $\sigma$ | 2.8644 | 2.1803 |
| $D_{increase}$ | 2.1825 | 5.6049 |
| $D_{max}$ | 2.3175 | 1 |
| $D_{decrease}$ | 9.0659 | 8.4948 |
| MLE | 7.46E+40 | 3.41E+45 |
| GOF | 0.981 | 0.9344 |
| AICc | -177.1496 | -197.6877 |
