# Supplementary material for: Evolutionary Game Theory and Social Learning Can Determine How Vaccine Scares Unfold
Source: PLoS Comput Biol. 2012 Apr 5;8(4):e1002452. doi: 10.1371/journal.pcbi.1002452 (PMC3320575; doi:10.1371/journal.pcbi.1002452)
Supplement: Table S6 — Estimated parameter values from bootstrapping for behavior-incidence model for Pertussis. Values represent median (median −2 standard deviations, median +2 standard deviations) from 50 bootstrap samples. (PDF) [file pcbi.1002452.s027.pdf]

t_{fit}	$D_{recovery}$	κ	σ	$r_{v,pre}$ x 10^{-4}	weighted error
1975	1.7 (-10, 14)	2.2 (-0.3, 4.7)	27 (-40, 94)	1.6 (0.8, 2.4)	0.02 (-0.04, 0.07)
1976	1.6 (1.3, 2.0)	1.2 (0.06, 2.4)	42 (-10, 94)	1.7 (-2.5, 5.9)	0.02 (-0.05, 0.08)
1977	2.8 (1.3, 4.4)	0.6 (-0.3, 1.6)	35 (-17, 88)	2.9 (-4.6, 10.5)	0.03 (-0.01, 0.07)
1978	4.2 (2.6, 5.8)	1.3 (0.4, 2.2)	20 (3.6, 37)	1.6 (-0.7, 4.0)	0.04 (0.001, 0.07)
1979	4.3 (2.8, 5.9)	1.3 (-0.1, 2.6)	22 (10, 34)	1.6 (0.4, 2.8)	0.04 (0.02, 0.06)
1980	3.9 (2.3, 5.5)	1.2 (0.04, 2.3)	25 (11, 39)	1.5 (0.7, 2.3)	0.03 (0.01, 0.05)
1981	3.6 (2.6, 4.6)	1.2 (0.6, 1.7)	26 (12, 40)	1.5 (1.2, 1.8)	0.04 (0.01, 0.06)
1982	3.6 (3.0, 4.3)	1.3 (1.0, 1.6)	24 (14, 33)	1.6 (1.4, 1.8)	0.04 (0.01, 0.06)
1983	3.5 (2.9, 4.1)	1.3 (1.1, 1.5)	24 (16, 32)	1.6 (1.4, 1.7)	0.03 (0.01, 0.05)
1984	3.6 (2.9, 4.2)	1.3 (1.1, 1.5)	24 (17, 31)	1.6 (1.4, 1.7)	0.04 (0.02, 0.06)
1985	3.3 (2.7, 3.9)	1.2 (1.0, 1.4)	26 (19, 33)	1.5 (1.4, 1.6)	0.03 (0.02, 0.05)
1986	3.1 (2.5, 3.8)	1.2 (1.0, 1.4)	29 (20, 37)	1.5 (1.4, 1.6)	0.04 (0.02, 0.05)
1987	3.0 (2.4, 3.6)	1.2 (1.0, 1.4)	29 (21, 37)	1.5 (1.4, 1.6)	0.04 (0.02, 0.05)
1988	3.0 (2.4, 3.6)	1.2 (1.1, 1.4)	27 (19, 35)	1.6 (1.5, 1.7)	0.04 (0.03, 0.06)

* Values represent median (median – 2 standard deviations, median + 2 standard deviations) from 50 bootstrap samples
