# Supplementary material for: Evolutionary Game Theory and Social Learning Can Determine How Vaccine Scares Unfold
Source: PLoS Comput Biol. 2012 Apr 5;8(4):e1002452. doi: 10.1371/journal.pcbi.1002452 (PMC3320575; doi:10.1371/journal.pcbi.1002452)
Supplement: Table S7 — Estimated parameter values from bootstrapping for behavior-incidence model for MMR. Values represent median (median −2 standard deviations, median +2 standard deviations) from 50 bootstrap samples. (PDF) [file pcbi.1002452.s028.pdf]

t_{fit}	$D_{recovery}$	κ	σ	$r_{v,pre}$ x 10^{-4}	δ (years)	weighted error x 10^{-4}
1997	11.9 (4.6, 19.3)	3.9 (1.9, 5.8)	17.3 (8.9, 25.8)	1.0 (0.5, 1.4)	1.8 (-2.1, 5.8)	0.0 (-0.0, 0.0)
1998	10.2 (2.3, 18.1)	1.1 (-0.4, 2.7)	14.0 (4.6, 23.4)	1.3 (0.7, 1.8)	6.1 (0.5, 11.8)	0.1 (-0.2, 0.4)
1999	0.9 (-8.0, 9.8)	2.3 (-0.6, 5.3)	14.3 (-3.7, 32.2)	1.0 (0.5, 1.6)	3.1 (-1.8, 7.9)	0.5 (-0.6, 1.5)
2000	0.9 (-4.7, 6.5)	2.2 (0.1, 4.2)	13.2 (-13.0, 39.5)	1.0 (0.7, 1.4)	3.1 (-0.2, 6.3)	0.6 (-0.6, 1.7)
2001	10.2 (-8.3, 28.7)	0.7 (-0.4, 1.8)	21.5 (-4.7, 47.7)	0.5 (-0.3, 1.3)	2.5 (-2.2, 7.2)	3.1 (0.4, 5.8)
2002	15.6 (-3.4, 34.6)	0.7 (-1.6, 3.1)	19.9 (-6.4, 46.2)	0.5 (-0.0, 1.0)	2.2 (-1.1, 5.5)	3.2 (0.5, 5.8)
2003	13.6 (-3.3, 30.4)	0.9 (-1.6, 3.4)	11.3 (-7.8, 30.5)	0.7 (0.3, 1.2)	1.7 (-2.7, 6.0)	2.9 (-0.7, 6.4)
2004	10.6 (6.4, 14.9)	1.3 (-1.0, 3.6)	7.4 (-5.7, 20.5)	0.7 (0.3, 1.1)	1.3 (-0.8, 3.4)	3.3 (0.4, 6.3)
2005	8.2 (7.2, 9.2)	3.4 (2.7, 4.1)	3.4 (1.4, 5.5)	0.9 (0.8, 1.0)	1.3 (1.0, 1.6)	3.9 (1.5, 6.4)
2006	7.7 (5.5, 10.0)	3.5 (0.7, 6.3)	3.4 (2.1, 4.7)	0.9 (0.8, 1.0)	1.4 (0.4, 2.3)	6.4 (0.6, 12.3)
2007	7.2 (6.4, 8.1)	3.3 (2.8, 3.8)	3.6 (2.8, 4.4)	0.9 (0.8, 1.0)	1.5 (0.9, 2.0)	7.2 (4.4, 10.0)
2008	7.4 (6.7, 8.1)	3.4 (3.1, 3.7)	3.5 (2.9, 4.2)	0.9 (0.8, 1.0)	1.3 (0.7, 1.9)	6.7 (3.7, 9.6)
2009	7.1 (6.6, 7.7)	3.4 (3.0, 3.8)	3.9 (3.1, 4.6)	0.9 (0.8, 1.0)	1.2 (0.6, 1.8)	9.1 (2.0, 16.2)

* Values represent median (median – 2 standard deviations, median + 2 standard deviations) from 50 bootstrap samples
