# Supplementary material for: Evolutionary Game Theory and Social Learning Can Determine How Vaccine Scares Unfold
Source: PLoS Comput Biol. 2012 Apr 5;8(4):e1002452. doi: 10.1371/journal.pcbi.1002452 (PMC3320575; doi:10.1371/journal.pcbi.1002452)
Supplement: Text S1 — Methods. (PDF) [file pcbi.1002452.s029.pdf]

SUPPORTING INFORMATION

'Evolutionary game theory and social learning can determine how vaccine scares unfold'

C.T. Bauch and S. Bhattacharyya

Item	Page
Supporting Text: Methods	3
Supporting Figure 1: Schematic diagram of risk evolution curves	11
Supporting Figure 2: Parsimony analysis of behavioral model, pertussis	12
Supporting Figure 3: Parsimony analysis of behavioral model, MMR	13
Supporting Figure 4: Parsimony analysis of behavior-incidence model, pertussis	14
Supporting Figure 5: Parsimony analysis of behavior-incidence model, MMR	15
Supporting Figures 6-11: Fit of behavioural model to correlated white noise data sets for risk evolution curves 1-6.	16
Supporting Figures 12-17: Fit of behaviour-prevalence model to correlated white noise data sets for risk evolution curves 1-6.	22
Supporting Figure 18: Predictive analysis of behavior-incidence model, pertussis, under Probabilistic sensitivity analysis (PSA)	28
Supporting Figure 19: Predictive analysis of behavior-incidence model, pertussis, under bootstrapping	32
Supporting Figure 20: Predictive analysis of behavior-incidence model, MMR, under Probabilistic sensitivity analysis (PSA)	36
Supporting Figure 21: Predictive analysis of behavior-incidence model, MMR, under bootstrapping	40
Supporting Table 1: Confidence intervals from bootstrapping for behavioral model (with social learning and feedback), pertussis and measles	44
Supporting Table 2: Fitting results for behavioral model (with social learning and feedback), pertussis and measles	45
Supporting Table 3: Fitting results for reduced behavioral model (with social learning but no feedback), pertussis and measles	46

Supporting Table 4: Fitting results for reduced behavioral model (with feedback but no social learning), pertussis and measles	47
Supporting Table 5: Fitting results for reduced behavioral model (with no social learning and no feedback), pertussis and measles	48
Supporting Table 6: Best-fit parameter values and confidence intervals from bootstrapping for behavior-incidence model, pertussis	49
Supporting Table 7: Best-fit parameter values and confidence intervals from bootstrapping for behavior-incidence model, MMR	50

Supporting Text

Behavioral models. The imitation dynamic assumes that an individual samples others in the population at some constant rate *s* and switches to the other person's strategy (if it differs from the individual's current strategy) with a probability proportional to the different in payoff between the two strategies, ΔE. The payoff to vaccinate will be taken as

$$E_v = B - c_v \tag{1}$$

where $B >> c_v$ represents a baseline payoff corresponding to a state of perfect health and c_v is the penalty for being vaccinated. Note that we assume a perfect vaccine. The payoff not to vaccinate will be taken as

$$E_n = B - c_i mL \tag{2}$$

where c_i is the penalty for being infected, *m* is a proportionality constant governing the probability of infection (with $B >> c_i m$), and *L* is the number of case notifications at time *t*, taken from the data in Figure 1 in the case of the behavioral model (hence, we assume these case notification data reflect the actual disease incidence experienced by the population up to a scaling factor). Equation (2) also represents that individuals are using a 'rule of thumb' to determine their probability of being infected, i.e., they assume it is simply linearly proportional to the current incidence of infected individuals in the population. The payoff gain for a vaccinator switching to a nonvaccinator strategy is therefore

$$\Delta E_{nv} = E_n - E_v = c_v - c_i mL \tag{3}$$

Hence, if *x* is the proportion of vaccinators in the population at time *t*, it means a vaccinator will encounter nonvaccinators at a rate *s*(1-*x*) (since they sample others at rate *s* and a proportion 1-*x* of their encounters will be with a nonvaccinators). Since there are *x* total vaccinators and the payoff gain is as in Equation (3), the total rate at which individuals switch from vaccinator to nonvaccinator is

$$\begin{cases} sx(1-x)\theta(c_v - c_i mL) & \text{when } \Delta E_{nv} = c_v - c_i mL > 0 \\ 0 & \text{when } \Delta E_{nv} = c_v - c_i mL \leq 0 \end{cases} \tag{4}$$

where θ is the proportionality constant from the probability of switching strategies being proportional to the payoff gain. Similarly, the total rate at which individuals switch from nonvaccinator to vaccinator is

$$\begin{cases} s(1-x)x\theta(-c_v + c_i mL) & \text{when } \Delta E_{vn} = -c_v + c_i mL > 0 \\ 0 & \text{when } \Delta E_{vn} = -c_v + c_i mL \le 0 \end{cases} \tag{5}$$

where the payoff gain has a sign that is opposite that of Equation (3) since the strategy switch is in the reverse direction. Therefore the total rate of change in the number of vaccinators *x* is the rate at which nonvaccinators become vaccinators—Equation (5)—minus the rate at which vaccinators become nonvaccinators—Equation (4)—which yields

$$\frac{dx}{dt} = s\theta x(1-x)(-c_v + c_i mL) \tag{6}$$

Using the substitutions

$$\kappa = s\theta c_i m \tag{7}$$

and

$$\omega = c_v / mc_i \tag{8}$$

this reduces to

$$\frac{dx}{dt} = \kappa x(1-x)(-\omega + L)\ , \tag{9}$$

which is the behavioral model with both social learning and feedback. The parameter ω has absorbed c_v which, unlike other parameters, evolves over time with the perceived vaccination penalty. Hence $\omega = \omega(t)$ is the risk evolution curve. Equation (9) is the form we use for our parsimony analysis.

The simplest possible reduced behavioral model with social learning but no feedback can be obtained by rewriting Eq. (9) as $dx/dt = x(1-x)(-\kappa\omega(t) + \kappa L)$ $= x(1-x)(-\omega'(t) + \kappa L)$, removing κL (since there is no feedback), and rewriting $\omega'(t)$ as $\omega(t)$ for simplicity, yielding:

$$\frac{dx}{dt} = x(1-x)(-\omega(t)) \tag{10}$$

which captures how coverage x decreases ($dx/dt<0$) when perceived vaccine risk is higher ($\omega(t)>0$). Note that ω(t) in this formulation can be positive or negative depending on whether vaccinating or not vaccinating is favoured at a given time.

The simplest possible reduced behavioral model with feedback but no social learning is:

$$x(t) = \rho L(t) - \omega(t) \tag{11}$$

which captures how x increases directly as ω decreases or as $L(t)$ increases.

Finally, the simplest possible reduced behavioral model with neither feedback nor social learning is just:

$$x(t) = 1 - \omega(t) \tag{12}$$

which captures how x increases as ω decreases. In this case, vaccine coverage tracks the inverse of perceived vaccine risk (penalty).

Risk evolution curves. A diagram of $\omega(t)$ appears in Supplementary Figure 9: $\omega(t)$ is ω_{pre} before the scare, climbs linearly for $D_{increase}$ years to reach a maximum of $\sigma\omega_{pre}$, and remains there for D_{max} years before declining linearly back to ω_{pre} over $D_{decrease}$ years. The five curves are #1 (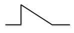 ;$D_{increase} = D_{max} = 0$, fit ω_{pre}, σ, $D_{decrease}$); #2 (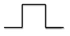 ; $D_{increase} = D_{decrease} = 0$, fit ω_{pre}, σ, D_{max}); #3 (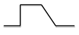 ; $D_{increase} = 0$, fit ω_{pre}, σ, $D_{decrease}$, D_{max}); #4 (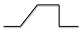 ; $D_{decrease} = 0$, fit ω_{pre}, σ, $D_{increase}$, D_{max}); #5 (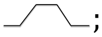; fit ω_{pre}, σ, $D_{decrease}$, $D_{increase}$, D_{max}).

Parsimony analysis for behavioral model. We fitted models (9)-(12) to vaccine coverage data in MATLAB R2008a. We used a trust-region reflective algorithm (a nonlinear constrained optimization method).[1] We computed the maximum likelihood estimator as $M = e^{-n/2} \Big/ \left(2\pi RSS_{vac}/n\right)^{n/2}$, where RSS_{vac} is the residual sum of squares of the fit between data and model and n is the number of data points.[2] To evaluate parsimony we used a modification of the Aikaike Information Criterion

intended for smaller datasets given by $AICc = -2\ln(M) + 2l + \left(2l(l+1)\right)/(n - l - 1)$, where l is the number of fitted parameters.[3] Compared to conventional AIC, the AICc imposes a larger penalty for more parameters. The model with the lowest AICc is considered the most parsimonious. To obtain 95% confidence intervals for the behavioral model with social learning and feedback, we adopted a non-parametric bootstrap approach that involves resampling the residuals obtained from fitting the model to the vaccine coverage data. We drew 1,000 bootstrap samples with replacement in each case and fit the model to each sample and thus obtained the bootstrap $G_\theta(t)$ distribution of the estimator θ. Then the exact upper (1 – α) confidence limit for θ is the value $t^*_{1-\alpha}$ such that $G_\theta(t^*_{1-\alpha})=1-\alpha$. Confidence intervals appear in supporting material.

Behavior-incidence model. Equation (9) was augmented with transmission dynamics to become[4]

$$\begin{aligned} \frac{dS}{dt} &= \mu(1-\varepsilon x) - \mu S - \beta SI - \tau S \\ \frac{dI}{dt} &= -\mu I + \beta SI - \gamma I + \tau S \\ \frac{dx}{dt} &= \kappa x(1-x)(-\omega + I) \end{aligned} \tag{13}$$

where S is the proportion susceptible, I is the proportion infectious, x is the proportion that are vaccinators, μ is the birth and death rate, ε the vaccine efficacy, β is the transmission rate, γ is the recovery rate, and τ is the case import rate. The proportion recovered/immune is 1-S-I. In the $x(t)$ equation, $L(t)$ has been replaced by $I(t)$. The financial cost of the vaccine could be subsumed in the parameter, c_i but vaccine costs are not relevant in this case since both vaccines were freely available through public health. Parents of infants are actually the decision-makers but we assume that parents always maximize their children's health. Initial conditions were $S(0)$=0.05, $I(0)$=0.0001, $x(0)$=0, and $t(0)$=1850. We set x=0.8 in 1946 for pertussis (1965 for measles) to represent vaccine introduction. The baseline

parameter values for pertussis were $1/\gamma$=22 days, R_0=17 (from which $\beta \approx R_0\gamma$), ε=1, μ=0.02/yr, and τ=3.7x10^{-6}/yr.[5-7] The baseline values for measles were the same except $1/\gamma$=13 days and ε=0.9.[5-7] Measles transmission varied seasonally according to $\beta(t)=b_0(1 + b_1\cos(t))$ with b_1=0.25/yr and b_0= $R_0\gamma$.[5-7] A delay was introduced for measles, modifying the $x(t)$ equation to become

$$dx/dt = \kappa x(1-x)(-\omega + I(t-\delta)) \tag{14}$$

Epidemiological parameters were varied in PSA.

<u>**Parsimony analysis for behavior-incidence model.**</u> Numerical code was written in C. Equation (13) was solved using a fixed stepsize fourth-order Runge-Kutta algorithm (an adaptive stepsize algorithm was found not to converge). To search the parameter space for the best fitting solutions, a shotgun hill-climbing algorithm [8] with adaptively shrinking search areas was used. We used the same maximum likelihood estimator as for the behavioral model. The parameter κ was fitted, as were parameters according to which curve was used, and also δ in the case of MMR. For the model with feedback but no social learning using the transmission model, the last line of equation (11) becomes:

$$x(t) = \rho I(t) - \omega(t) \tag{15}$$

We used the same AICc measure as for the behavioral model.

Predictive analysis of behavior-incidence model. The model was fitted as described above, except the RSS was a weighted sum of the RSS for both vaccine coverage and disease incidence. The residual sum of squares for vaccine coverage was computed as

$$RSS_{vac} = \sum_{i=1}^{n}(C_i - x_i)^2 \tag{16}$$

where C_i is the vaccine coverage in year i in the data and x_i is the corresponding vaccine coverage in year i in the model. Before computing the residual sum of squares for disease incidence, we normalized the disease incidence for both model and in the data according to:

$$D_i = \frac{d_i}{\sum_{i=1}^{n} d_i} \tag{17}$$

where d_i is the raw disease incidence in year *i* before normalization and D_i is the normalized disease incidence. This ensured that disease incidence in model and data could be compared at the same scale. The residual sum of squares for disease incidence was then computed as

$$RSS_{inc} = \sum_{i=1}^{n} \left(D_i^{\text{model}} - D_i^{\text{data}} \right)^2 \tag{18}$$

where D_i^{model} is the normalized disease incidence in year *i* in the model and D_i^{data} is the normalized disease incidence in year *i* in the data. To fit the data, we then minimized the combined weighted error

$$E = RSS_{vac} + wRSS_{inc} \tag{19}$$

where *w* is the weight parameter. We used *w*=1 for pertussis and *w*=0.01 for MMR. The lower value for MMR is due to less information provided by case notification time series compared to the pertussis vaccine scare. We varied *w* in PSA.

PSA for behavior-incidence model. PSA was conducted using the following ranges—pertussis: R_0 [14,20], γ [1/25,1/19], ε [0.9, 1], τ [0, 3.7x10^{-5}], S(0) [0.025, 0.075], I(0) [0.00005, 0.00015], *w* [0.5, 1.5]; measles: R_0 [14,20], γ [1/15,1/11], ε [0.85, 0.95], τ [0, 3.7x10^{-5}], b_1 [0.15, 0.35], S(0) [0.025, 0.075], I(0) [0.00005, 0.00015], *w* [0.001, 0.05]. 50 samples were drawn from triangular distributions based on these parameter ranges. For each sample, the model was fitted as before.

Bootstrapping for behavior-incidence model. The same bootstrapping method was used for the behavior-incidence model as for the behavioral model, except that both best-fitting incidence and vaccine coverage time series were resampled. 50 bootstrap samples were generated and simulated. For each sample, the model was fitted as before.

Analysis using correlated white noise. The AIC analysis for the behavioural model and behaviour-prevalence model was repeated using correlated white noise data instead of the empirical MMR vaccine coverage data. Correlated white noise were generated according to the following algorithm:

$$
\begin{aligned}
C_1 &= 0.6 + 0.4 \times rand \\
C_2 &= 0.5 \times (0.6 + 0.4 \times rand) + 0.5 \times (C_1) \\
C_3 &= 0.5 \times (0.6 + 0.4 \times rand) + 0.5 \times (C_2) \\
&\vdots \\
C_n &= 0.5 \times (0.6 + 0.4 \times rand) + 0.5 \times (C_{n-1})
\end{aligned}
$$

where *rand* is a random number between 0 and 1 generated from uniform distribution and where C_k is the vaccine coverage in year k of the generated white noise time series. Correlation between successive steps ensures that the generated time series appears more smooth than a pure white noise time series would, and hence is a stronger test of the behaviour-prevalence model. The analysis was carried out for risk evolution curves #1-#5, but also for completely flat risk evolution curve corresponding to no vaccine scare (σ=1), denoted risk evolution curve #6 (since the correlated white noise data by definition do not exhibit the same secular trend that vaccine coverage data would during a vaccine scare). For each risk evolution curve, we present figures of the best fitting model to the white noise data as well as the AICc score and the natural logarithm of the maximum likelihood function (for the behaviour-prevalence model) or the goodness-of-fit (for the behavioural model), compared to the same for the best fitting model to the empirical MMR vaccine coverage data.

Discussion of model selection exercise. By adding a sufficient number of free parameters to the risk evolution curve it will always be possible to achieve an arbitrarily good AICc score without adding social learning or feedback. The AICc equation is $AICc = -2\ln(M) + 2l + \left(2l(l+1)\right)/\left(n - l - 1\right)$ where ln(M) is the logarithm of

the maximum likelihood estimator M, l is the number of free parameters, and n is the number of data points. Vaccine coverage data are reported only to two decimal places. Hence, a risk evolution curve that matches the data exactly could be defined using n-2 free parameters, where a first parameter is assigned to two time points with identical vaccine coverage, a second parameter is assigned to another two time points with identical vaccine coverage, and the remaining n-4 parameters are assigned to the remaining n-2 time points. This would cause $\ln(M)$ to become infinite while the second and third terms remain of order n^2. In intermediate cases, it seems likely that we should always reach a point where enough free parameters have been added to allow a "naked" risk evolution curve to outperform the corresponding behavior-incidence model: in the current analysis that point was reached with curve #5 with its five free parameters.

References

1. Byrd RH, Schnabel RB, Schultz GA. A trust region algorithm for nonlinearly constrained optimization. SIAM J Numer Anal 1987;24:1152-70.
2. Lalam N, Klaassen CAJ. Pseudo maximum likelihood estimation for differential equations. EURANDOM Preprint. 2006;2006-018.
3. Burnham KP, Anderson DR. Model selection and multimodel inference: a practical information-theoretic approach, 2nd ed. : Springer-Verlag; 2002.
4. Bauch CT. Imitation dynamics predict vaccinating behaviour. Proc R Soc Lond B. 2005;272:1669-75.
5. Bauch C, Earn D. Transients and attractors in epidemics. Proc Roy Soc Lond A. 2003;270:1573-8.
6. Earn D, Rohani P, Bolker B, Grenfell B. A simple model for complex dynamical transitions in epidemics. Science. 2000;287:667-70.
7. Rohani P, Earn D, Grenfell B. Opposite patterns of synchrony in sympatric disease metapopulations. Science. 1999;286:968-71.
8. Jacobsen SH, Yucesan E. Analyzing the performance of generalized hill-climbing algorithms. J Heuristics. 2004;10:387-405.
